# Gut microbiota composition is associated with the efficacy of Delta-24-RGDOX in malignant gliomas

**DOI:** 10.1016/j.omton.2024.200801

**Published:** 2024-04-17

**Authors:** Natalie M. Meléndez-Vázquez, Teresa T. Nguyen, Xuejun Fan, Andrés R. López-Rivas, Juan Fueyo, Candelaria Gomez-Manzano, Filipa Godoy-Vitorino

## Main text

(Molecular Therapy: Oncology *32*, 200787; March 2024)

In the originally published version of this article, we, the authors, stated in the summary and introduction that there were no studies linking the gut microbiota with viroimmunotherapy efficacy. However, we realized that a study published in October 2023—at the same time this article was submitted—reported having supplemented animals with *Bifidobacterium* during viroimmunotherapy against melanoma. Although the studies are different, and we report on the naturally occurring Bifidobacteria in animals that responded to the therapy (not on supplementation), we decided to be more specific on the results of our particular glioblastoma model.

The summary and introduction of the original article have been corrected to reflect this change, and the authors apologize for any confusion this may have caused.

