# Supplementary material for: Gut microbiota composition is associated with the efficacy of Delta-24-RGDOX in malignant gliomas
Source: Mol Ther Oncol. 2024 Feb 28;32(1):200787. doi: 10.1016/j.omton.2024.200787 (PMC10951704; doi:10.1016/j.omton.2024.200787)
Supplement: Document S1. Figures S1‒S3 and Tables S1–S6 [file mmc1.pdf]

OMTON, Volume *32*

Supplemental information

Gut microbiota composition is associated with the efficacy of Delta-24-RGDOX in malignant gliomas

Natalie M. Meléndez-Vázquez, Teresa T. Nguyen, Xuejun Fan, Andrés R. López-Rivas, Juan Fueyo, Candelaria Gomez-Manzano, and Filipa Godoy-Vitorino

Supplemental Information

Table S1: Experimental groups, number of animals and summary of the number of sequences (reads) and ASVs across the GSC-005 glioblastoma cohort dataset.

	Experimental Group	Samples	Mice Survival Period	Mean Survival (days) ± Stdev*	Average Number of Reads ± Stdev*	Average Number of ASVs ± Stdev*
GSC-005 Glioblastoma Cohort	Naive	5	NA	NA	19432.00 ± 11259.68	209.00 ± 34.31
	PBS	3	50 - 56 days	51.33 ± 1.53	19725.00 ± 10266.22	290.67 ± 93.07
	Indoximod	5	50 - 56 days	53.40 ± 2.07	10415.20 ± 2436.14	203.60 ± 15.47
	Delta-24-RGDOX	4	More than 100 days	168.00 ± 39.23	27508.50 ± 14201.95	219.75 ± 68.65
	**TOTAL**	17				

*Stdev: standard deviation

Table S2: Experimental groups, number of animals and summary of the number of sequences (reads) and ASVs across the GL261-5 glioblastoma cohort dataset.

	Experimental Group	Samples	Mean Survival (days) ± Stdev*	Average Number of Reads ± Stdev*	Average Number of ASVs ± Stdev*
**GL261-5 Glioblastoma Cohort**	PBS	5	43.00 ± 6.00	15,536.80 ± 1417.51	176.80 ± 26.25
	Delta-24-RGDOX + Indoximod + αCD4	5	53.0 ± 18.60	14,807.20 ± 2098.82	180.60 ± 30.32
	Delta-24-RGDOX + Indoximod + IgG	5	100.0 ± 0.00	13,857.40 ± 1426.24	184.00 ± 18.68
	**TOTAL**	15			

*Stdev: standard deviation

Table S3: Statistical values of Tukey's Multiple Comparisons Test on the median survival of both GSC-005 and GL261-5 GBM cohorts.

	Experimental Group 1	Experimental Group 2	PERMANOVA p-value
**GSC-005 GBM Cohort**	PBS (51.33 days ± 1.53) (n=3)	Indoximod (53.40 days ± 2.07) (n=5)	0.9915
	PBS (51.33 days ± 1.53) (n=3)	Delta-24-RGDOX (168.00 days ± 39.23) (n=4)	**0.0002**
	Indoximod (53.40 days ± 2.07) (n=5)	Delta-24-RGDOX (168.00 days ± 39.23) (n=4)	**<0.0001**
**GL261-5 GBM Cohort**	PBS (43.00 days ± 6.00) (n=5)	Delta-24-RGDOX + Indoximod + αCD4 (53.0 days ± 18.60) (n=5)	0.371
	PBS (43.00 days ± 6.00) (n=5)	Delta-24-RGDOX + Indoximod + IgG (100.0 days ± 0.00) (n=5)	**<0.0001**
	Delta-24-RGDOX + Indoximod + αCD4 (53.0 days ± 18.60) (n=5)	Delta-24-RGDOX + Indoximod + IgG (100.0 days ± 0.00) (n=5)	**<0.0001**

Significant p-values in bold.

Table S4: Statistical values of PERMANOVA and Kruskal-Wallis (KW) pairwise tests on the compositional biplots and alpha diversity metrics for the main text Figure 2.

			Figure 2A	Figure 2B	Figure 2C	Figure 2D
	**Experimental Group 1**	**Experimental Group 2**	**PERMANOVA p-value**	**PERMANOVA p-value**	**Chao1 KW p-value**	**Shannon KW p-value**
**GSC-005 GBM Cohort**	Naive (n=5)	PBS 50-56 days (n=3)	**0.022**	-	0.051	**0.025**
	Naive (n=5)	Indoximod 50-56 days (n=5)	**0.009**	-	0.753	0.076
	Naive (n=5)	Delta-24-RGDOX more than 100 days (n=4)	**0.007**	-	0.806	0.806
	PBS 50-56 days (n=3)	Indoximod 50-56 days (n=5)	**0.017**	**0.017**	0.101	**0.025**
	PBS 50-56 days (n=3)	Delta-24-RGDOX more than 100 days (n=4)	0.051	**0.032**	0.289	0.157
	Indoximod 50-56 days (n=5)	Delta-24-RGDOX more than 100 days (n=4)	0.139	0.059	1.000	0.462

Significant p-values in bold.

Table S5: Statistical values of the Wilcoxon Rank-Sum (WRST) pairwise test on the Firmicutes/Bacteroidetes ratio for the main text Figure 3.

	Experimental Group 1	Experimental Group 2	WRST p-value
**GSC-005 GBM Cohort**	Naive (n=5)	PBS 50-56 days (n=3)	0.21
	Naive (n=5)	Indoximod 50-56 days (n=5)	1.00
	Naive (n=5)	Delta-24-RGDOX more than 100 days (n=4)	1.00
	PBS 50-56 days (n=3)	Indoximod 50-56 days (n=5)	0.36
	PBS 50-56 days (n=3)	Delta-24-RGDOX more than 100 days (n=4)	0.91
	Indoximod 50-56 days (n=5)	Delta-24-RGDOX more than 100 days (n=4)	0.91

Significant p-values in bold.

Table S6: Statistical values of PERMANOVA, Kruskal-Wallis (KW) and Wilcoxon Rank-Sum (WRST) pairwise tests on the compositional biplots, alpha diversity metrics, and F/B ratios respectively for main text Figure 5.

			Figure 5A	Figure 5B		Figure 5C
	Experimental Group 1	Experimental Group 2	PERMANOVA p-value	Chao1 KW p-value	Shannon KW p-value	WRST p-value
GL261-5 GBM Cohort	PBS (n=5)	Delta-24-RGDOX + Indoximod + αCD4 (n=5)	0.055	0.754	0.917	0.67
	PBS (n=5)	Delta-24-RGDOX + Indoximod + IgG (n=5)	**0.015**	0.465	0.754	0.67
	Delta-24-RGDOX + Indoximod + αCD4 (n=5)	Delta-24-RGDOX + Indoximod + IgG (n=5)	0.265	0.917	0.754	0.67

Significant p-values in bold.

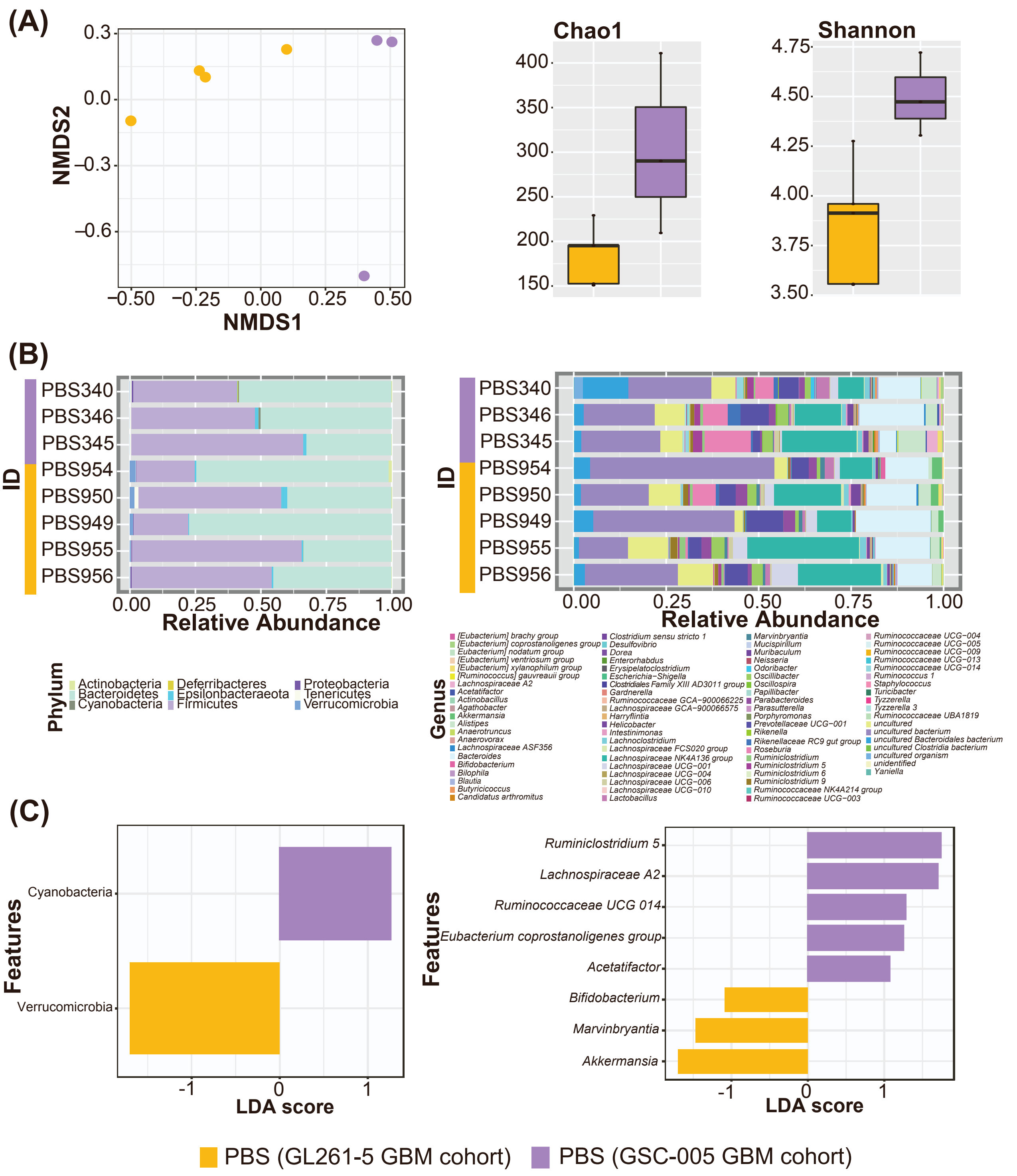

<u>Figure S1:</u> **(A)** Changes in bacterial composition and structure between the control groups (PBS administered mice) of the GL261-5 and GSC-005 glioma-bearing mice (ANOSIM p-value=0.016; stress value=9.572417e-05). Even though no differences in richness (Chao1) were observed (KW p-value=0.05), there is a tendency of a higher number of species in the PBS GSC-005 glioma-bearing mice. When evaluating the Shannon Index, we observed significant differences in the bacterial community diversity between both groups (KW p-value=0.03), observing a higher diversity in the PBS GSC-005 glioma-bearing mice. **(B)** Demonstrates the taxonomic changes at phylum and genus level between both groups. We observe a higher abundance of Verrucomicrobia in the GL261-5 glioma-bearing mice, while Cyanobacteria and Tenericutes is higher in GSC-005 glioma-bearing mice. **(C)** Highlights the biomarkers of each PBS group with a Linear Discriminant Analysis Effect Size (LeFSe) (Log LDA Score: 1.0; p-value cutoff: 0.05 Original). These differences highlight the importance of having separate microbiome analysis for both glioblastoma cohorts.

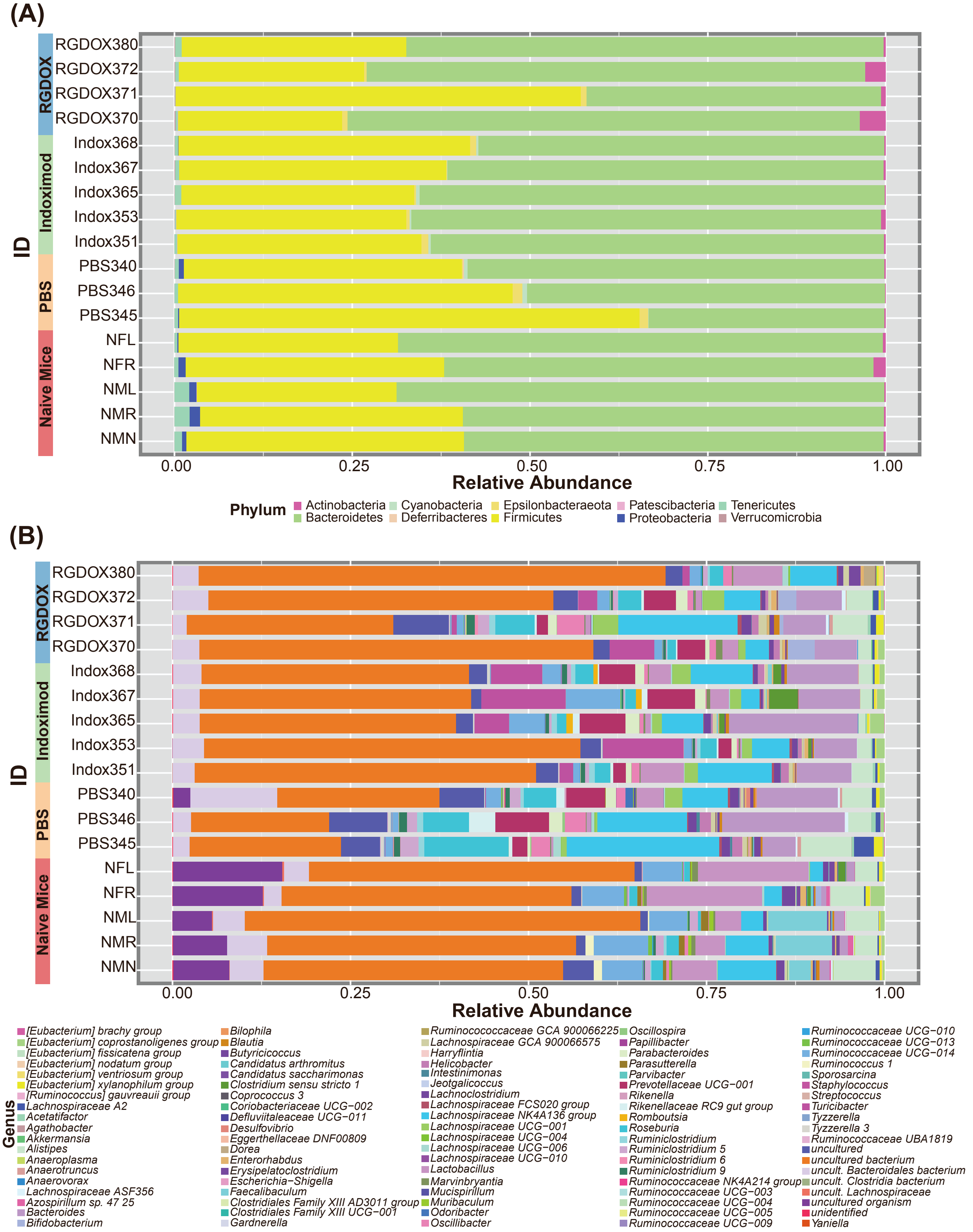

<u>Figure S2:</u> **Taxonomic composition of gut bacteria between groups showing that responders to oncolytic viral therapy that had longer survival periods have a gut microbiome profile similar to that of Naive mice.** Taxonomic changes are observed at phylum (A) and genus (B) level between groups. We observe a higher abundance of Actinobacteria in the oncolytic viral therapy mice responders, highlighted by an abundance of *Bifidobacterium*.

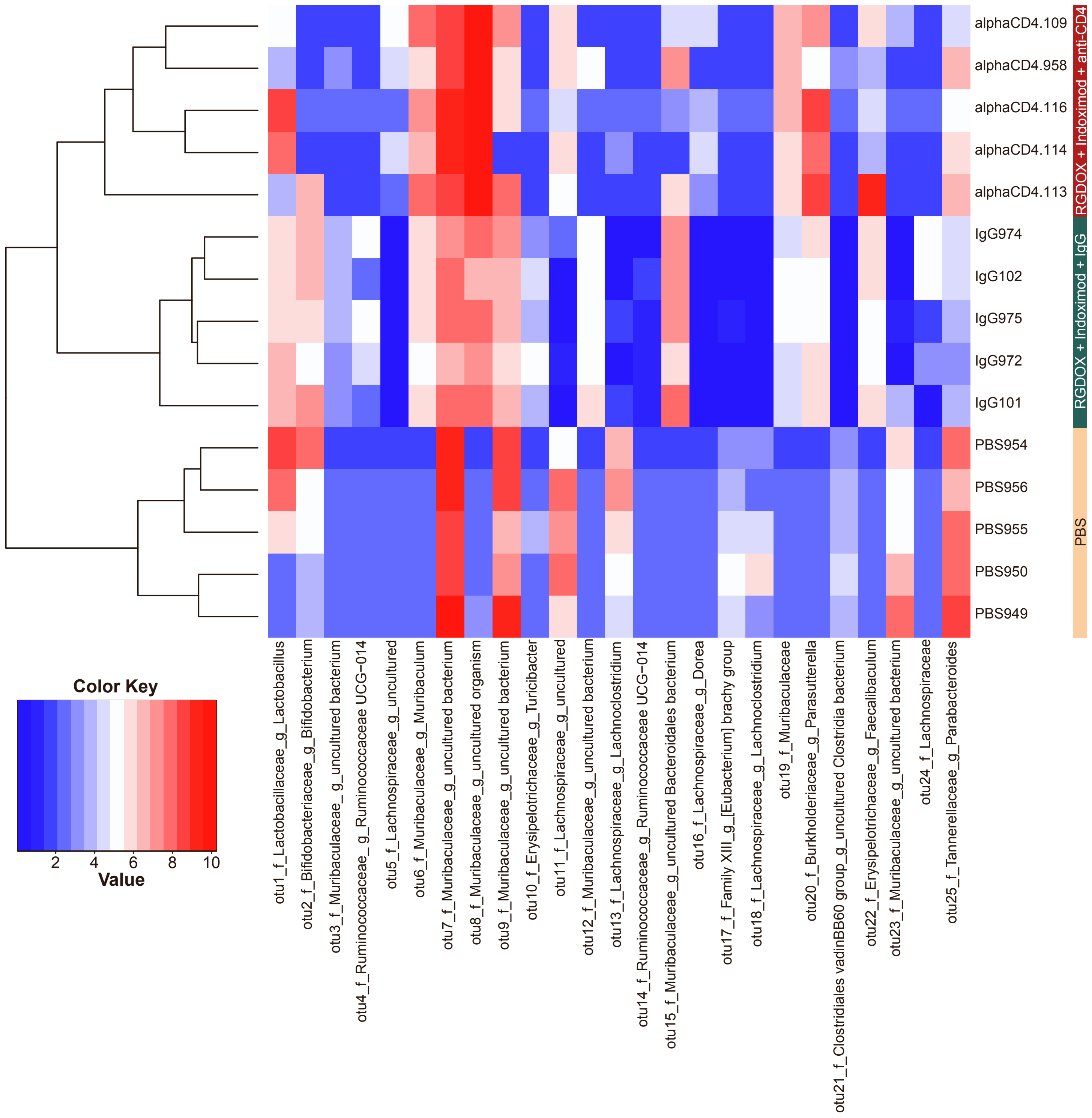

<u>Figure S3:</u> Heatmap of significant ASVs (raw p-value<0.01) from the GL261-5 cohort highlights the significant reduction of *Bifidobacterium* in the viroimmunotherapy and Indoximod-treated mice that had depleted $CD4^{+}$ T cells.
