# Supplementary material for: Gut microbiota composition is associated with the efficacy of Delta-24-RGDOX in malignant gliomas
Source: Mol Ther Oncol. 2024 Feb 28;32(1):200787. doi: 10.1016/j.omton.2024.200787 (PMC10951704; doi:10.1016/j.omton.2024.200787)
Supplement: Document S2. Article plus supplemental information [file mmc2.pdf]

Molecular Therapy
Oncology
Original Article

Gut microbiota composition is associated with the efficacy of Delta-24-RGDOX in malignant gliomas

Natalie M. Meléndez-Vázquez,[1] Teresa T. Nguyen,[2,3] Xuejun Fan,[2] Andrés R. López-Rivas,[2] Juan Fueyo,[2] Candelaria Gomez-Manzano,[2] and Filipa Godoy-Vitorino[1]

[1]Department of Microbiology and Medical Zoology, University of Puerto Rico, School of Medicine, Medical Sciences Campus, San Juan 00918 PR, USA; [2]Department of Neuro-Oncology, The University of Texas MD Anderson Cancer Center, Houston, TX 77030, USA

Glioblastoma, the most common primary brain tumor, has a 6.8% survival rate 5 years post diagnosis. Our team developed an oncolytic adenovirus with an OX-40L expression cassette named Delta-24-RGDOX. While studies have revealed the interaction between the gut microbiota and immunotherapy agents, there are no studies linking the gut microbiota with viroimmunotherapy efficacy. We hypothesize that gut bacterial signatures will be associated with oncolytic viral therapy efficacy. To test this hypothesis, we evaluated the changes in gut microbiota in two mouse cohorts: (1) GSC-005 glioblastoma-bearing mice treated orally with indoximod, an immunotherapeutic agent, or with Delta-24-RGDOX by intratumoral injection and (2) a mouse cohort harboring GL261-5 tumors used to mechanistically evaluate the importance of $CD4^+$ T cells in relation to viroimmunotherapy efficacy. Microbiota assessment indicated significant differences in the structure of the gut bacterial communities in viroimmunotherapy-treated animals with higher survival compared with control or indoximod-treated animals. Moreover, viroimmunotherapy-treated mice with prolonged survival had a higher abundance of *Bifidobacterium*. The $CD4^+$ T cell depletion was associated with gut dysbiosis, lower mouse survival, and lower antitumor efficacy of the therapy. These findings suggest that microbiota modulation along the gut-glioma axis contributes to the clinical efficacy and patient survival of viroimmunotherapy treated animals.

INTRODUCTION

Glioblastoma (GBM) is the most common and aggressive primary malignant brain tumor. The current standard of care, including radiotherapy, chemotherapy, and surgery, only extends the overall patient survival to approximately 15 months,[1–3,] with a 6.8% 5-year survival rate.[1] Upon recurrence, the median survival associated with GBM is typically 6 months.[4] While immunotherapy using immune checkpoint inhibitors (ICIs) has shown success in several solid tumors, such as melanoma,[5,6] and in preclinical studies on GBM murine models,[7–9] its effectiveness in patients with GBM has been limited.[10–12]

The development of oncolytic virus-based therapy has revealed encouraging results in preclinical and clinical settings.[13] Besides their direct oncolytic effect on tumor cells,[14,15] oncolytic viruses exert an immunostimulatory effect through activating cytokines and a T cell-mediated response, leading to the induction of antitumoral immunity.[16–18] Specifically, our group developed Delta-24-RGD (DNX-2401), a replication-competent adenovirus with tumor selectivity and enhanced infectivity.[16,19] A phase I clinical trial testing Delta-24-RGD on recurrent GBM patients extended survival by more than 3 years in 20% of patients, and 12% of responders had more than a 95% reduction in tumor size (ClinicalTrials.gov: NCT00805376).[20] Additionally, encouraging results have also been observed in two other clinical trials in adult and pediatric patients with malignant gliomas or diffuse intrinsic pontine gliomas (DIPGs) (ClinicalTrials.gov: NCT02798406 and NCT03178032, respectively).[21,22] To further improve the efficacy of Delta-24-RGD, we modified the agent to express the immune costimulatory OX40 ligand (OX40L), which is known to enhance tumor-specific T cell activation as well as the antigen-presenting capabilities of tumor cells.[23] Preclinical studies using this new oncolytic adenovirus, Delta-24-RGDOX, have shown that the indoleamine-2,3-dioxygenase (IDO)-dependent immunosuppressive pathways are responsible for mechanisms of resistance of solid tumors to virotherapy and that the combination of Delta-24-RGDOX with inhibitors of IDO results in better preclinical outcomes.[24]

Extensive literature has described the importance of the human gut microbiota, and its integration into the hallmarks of cancer reflects its importance to human health.[25] Several studies have described a close relationship between the gut microbiota and immunotherapy efficacy.[26–29] Specifically, a preclinical study with anti-PD-L1 treatment

Received 25 October 2023; accepted 26 February 2024;
https://doi.org/10.1016/j.omton.2024.200787.

[3]Present address: Immuno-Oncology, Regeneron Pharmaceuticals, Tarrytown, NY 10591, USA

Correspondence: Candelaria Gomez-Manzano, Department of Neuro-Oncology, The University of Texas MD Anderson Cancer Center, Houston, TX, USA.
E-mail: cmanzano@mdanderson.org
Correspondence: Filipa Godoy-Vitorino, Department of Microbiology and Medical Zoology, University of Puerto Rico, School of Medicine, Medical Sciences Campus, San Juan, PR 00918, USA.
E-mail: filipa.godoy@upr.edu

This is an open access article under the CC BY license (http://creativecommons.org/licenses/by/4.0/).

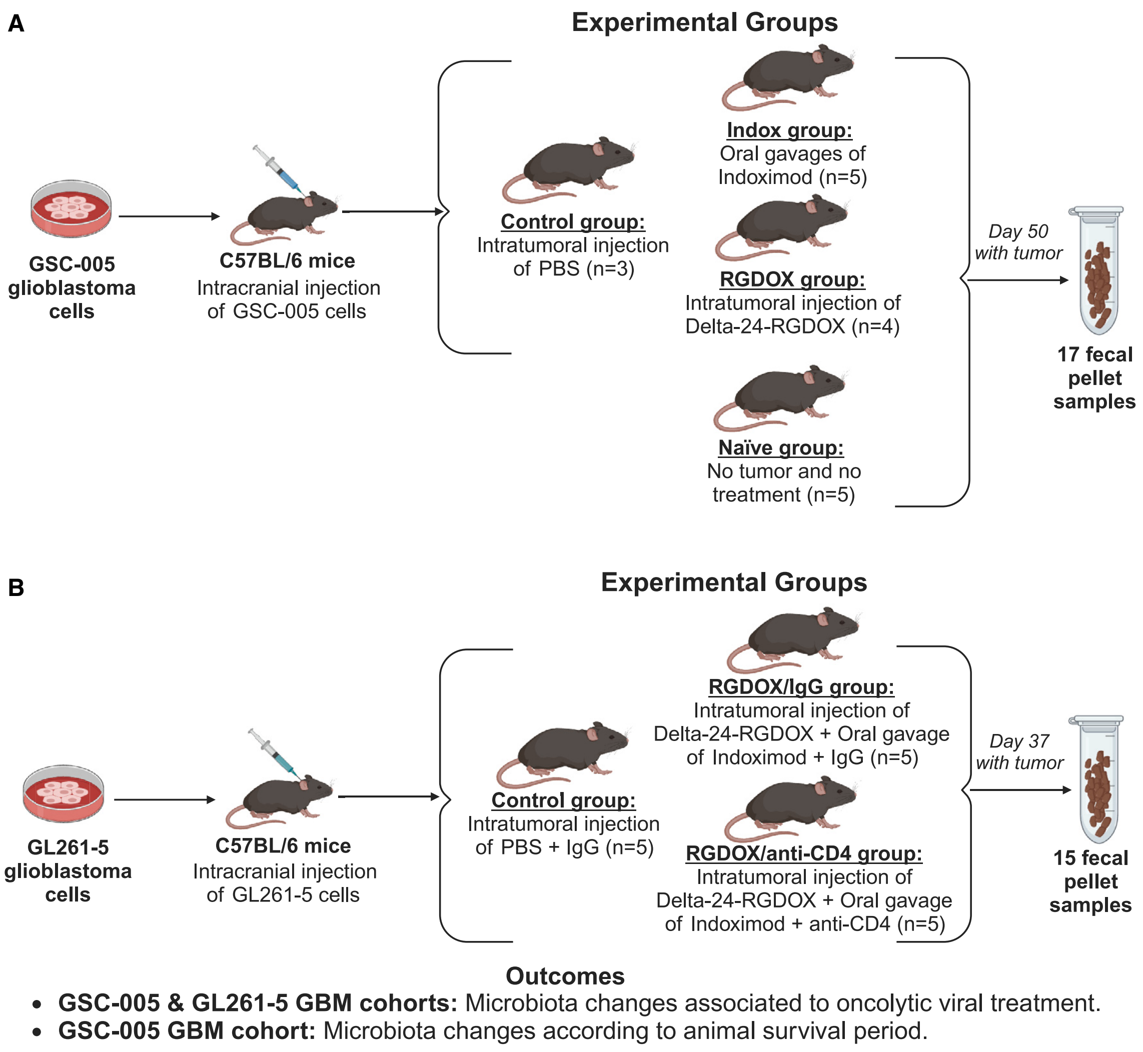

Figure 1. Conceptual framework of the GBM-microbiome study design

(A) The GSC-005 GBM cohort compromises four experimental groups: naive (n = 5), control (n = 3), indoximod treated (n = 5), and Delta-24-RGDOX treated (n = 4). (B) The GL261-5 GBM cohort with a total of three experimental groups: control (n = 5), Delta-24-RGDOX and indoximod treated plus the IgG control antibody (n = 5), and Delta-24-RGDOX and indoximod treated plus the CD4 neutralizing antibody (n = 5). Study design outcomes include microbiota changes related to viroimmunotherapy treatment for both cohorts and association with mouse survival period for the GSC-005 GBM cohort. Created with BioRender.

found an association between *Bifidobacterium* and antitumor T cell response.[27] Differential gut microbiota dynamics, including a higher gut diversity and an increase in Ruminococcaceae and *Faecalibacterium,* have been associated with melanoma patients responding to anti-PD-1.[30]

Understanding the gut-glioma axis might provide novel mechanisms of efficacy and resistance to therapies of solid tumors, including gliomas. To our knowledge, the association of the gut microbiota to the response to virotherapy has yet to be addressed. This study aims to determine whether the gut microbiota composition is linked to the efficacy of oncolytic viruses as single therapy or combined with IDO inhibitors in two murine glioma models. In addition, we aimed to understand the extent to which modification in the population of $CD4^+$ T cells contributes to changes in the gut microbiota. Our study demonstrates a strong positive relation between gut microbiota and viroimmunotherapy and suggests the possibility of modifying the gut-glioma axis to improve response to therapy.

RESULTS

To better understand the link between the gut microbiota and virotherapy, we collected fecal samples from two independent experimental studies to define bacterial composition and diversity associated with the therapy (Figure 1). In the first set of experiments, C57BL/6 mice were implanted intracranially with GSC-005 murine glioma cells and randomly assigned to receive treatment with

Delta-24-RGDOX or the immunomodulatory IDO inhibitor indoximod (Figure 1A).[24] A second set of experiments involved the implantation of GL261-5 murine glioma cells into the brain of C57BL/6 mice, followed by treatment with Delta-24-RGDOX in combination with indoximod in the context of CD4 T cell depletion to decipher their role in the gut microbiota and response to therapy (Figure 1B).[24]

Microbiota profiling using 16S ribosomal RNA (rRNA) gene sequencing was performed on fecal samples. After a detailed quality assessment of the 16S rRNA dataset, we recovered more than 77,000 good-quality reads for the GSC-005 GBM cohort (Table S1) and 44,000 for the GL261-5 GBM cohort (Table S2). The GSC-005 GBM cohort analysis used a rarefaction level of 6,448 reads per sample, and for the GL261-5 GBM cohort, we used 11,707 to guarantee that the even subsampling among all samples would be controlled for bias in the diversity estimates. As we found significant differences between the gut bacterial structure of PBS-treated GL261-5 and the PBS-treated GSC-005 (analysis of similarities [ANOSIM] p = 0.016; stress value = 9.572417e−05), we decided to analyze the data for these two cohorts independently (Figure S1).

Increase in anti-inflammatory gut biota, *Bifidobacterium* and *Akkermansia*, is associated with the viroimmunotherapy treatment

Animals treated with Delta-24-RGDOX exhibited significantly prolonged survival (168.00days ± 39.23) compared with indoximod- or PBS-treated mice (53.40 days ± 2.07 or 51.33 ± 1.53, respectively) (Tables S1 and S3). Prokaryotic community structure and composition were analyzed for both treatments (Delta-24-RGDOX and indoximod) and mouse survival period (50–56 days and more than 100 days) in the GSC-005 GBM cohort (Figures 2A and 2B). The healthy naive mice had a distinct bacterial composition compared with those bearing intracranial tumors and treated either with PBS (permutational multivariate analysis of variance [PERMANOVA] p = 0.022) or indoximod (PERMANOVA p = 0.009) (Figure 2A; Table S4). The taxa arrow-feature Muribaculaceae uncultured bacterium explains the distances formed by the naive group clustering (Figure 2A). Our results also indicate significant differences in the composition of the gut bacterial communities in Delta-24-RGDOX-treated animals with higher survival compared with the naive group (PERMANOVA p = 0.007) (Figure 2A; Table S4). Additionally, animals treated with indoximod had significant PERMANOVA distances to the PBS-treated animals (p = 0.017) (Figures 2A and 2B; Table S4). An in-depth analysis between the treatment groups, in which naive samples were removed, depicted significant differences between the animals treated with PBS and those with Delta-24-RGDOX (PERMANOVA p = 0.032) (Figure 2B; Table S4). Furthermore, though there were no significant differences in the richness on the gut microbiota among the different treatment groups (Figure 2C), we observed prominent changes in bacterial diversity between the naive and PBS-treated group (survival of 50–56 days) (Kruskal-Wallis [KW] p = 0.025347; Table S1), highlighting that the tumor presence is associated with changes in the gut biota (Figure 2D; Table S4). We also found differences in diversity between the gut microbiota of PBS- and indoximod-treated mice (both with survival of 50–56 days) (KW p = 0.025347; Figure 2D; Table S4). The highest gut diversity was observed in tumor-bearing control animals (PBS), emphasizing the impact tumor presence has on modulating bacterial diversity (Figure 2D; Table S4). We also found similarities in the alpha diversity levels between the indoximod- and viroimmunotherapy-treated mice compared with the naive controls (Figure 2D; Table S4). This indicates an effect of both immunomodulators, Delta-24-RGDOX and indoximod, on modifying the diversity associated with the tumor into one that is most similar to the "healthy" naive group.

Taxonomic profile analyses identified differences in composition at the phylum (Figure S2A) and genus (Figure S2B) levels among the gut microbiota from the experimental groups. The most drastic changes were an increased abundance in Actinobacteria among the viroimmunotherapy-treated animals (Figure S2A), represented by *Bifidobacterium* (Figure S2B). As the Firmicutes/Bacteroidetes (F/B) ratio is essential in maintaining normal gut homeostasis,[31] we analyzed this parameter among our samples. Our results showed that, although not statistically significant, samples from PBS-treated mice had a higher F/B ratio compared with the other groups (Wilcoxon rank-sum test [WRST] p > 0.05; Figure 3; Table S5), suggesting that the presence of the brain tumor induces gut dysbiosis. The Delta-24-RGDOX- and indoximod-treated animals had reduced F/B ratio, similar to those of the naive mice (WRST p > 0.05; Figure 3; Table S5). As both indoximod- and viroimmunotherapy-treated mice show alpha diversity and F/B ratios similar to those of the naive controls, it suggests that treatment with any of the two immunomodulators is associated with likely reversal of the tumor-associated dysbiosis.

A multivariable association analysis through MaAslin2 revealed that, for the naïve mice, there was a significantly higher dominance in the phylum Proteobacteria (false discovery rate [FDR] p = 1.486e−02) (Figure 4A) and the genera *Lactobacillus* (FDR p = 8.972e−02) (Figure 4B), *Muribaculum* (FDR p = 1.367e−02) (Figure 4C), and *Parasutterella* (FDR p = 1.348e−02) (Figure 4D). On the other hand, in the PBS-treated mice, the model identified a higher abundance of the phyla Epsilonbacteraeota (FDR p = 4.266e−02) (Figure 4E) and Cyanobacteria (FDR p = 1.910e−02) (Figure 4F) and the genera *Intestinimonas* (FDR p = 3.033e−02) (Figure 4G), *Oscillospira* (FDR p = 3.549e−02) (Figure 4H), *Ruminiclostridium* (FDR p = 4.862e−02) (Figure 4I), and *Roseburia* (FDR p = 1.348e−02) (Figure 4J). In the case of indoximod-treated animals, a higher abundance of *Turicibacter* was detected (FDR p = 2.000e−02) (Figure 4K). Additionally, in the viroimmunotherapy-treated mice, a significant association between enrichment of Actinobacteria (FDR p = 1.263e−01) (Figure 4L) and Verrucomicrobia (FDR p = 1.486e−02) was found (Figure 4M), characterized by increased presence of *Bifidobacterium* (FDR p = 9.642e−02) (Figure 4N) and *Akkermansia* (FDR p = 1.959e−02) (Figure 4O), respectively. In summary, these preliminary results suggest that (1) indoximod-treated mice have an increase in taxa capable of deconjugating bile acids (*Turicibacter*),[32] and (2) animals that responded to the viroimmunotherapy showed an increase in

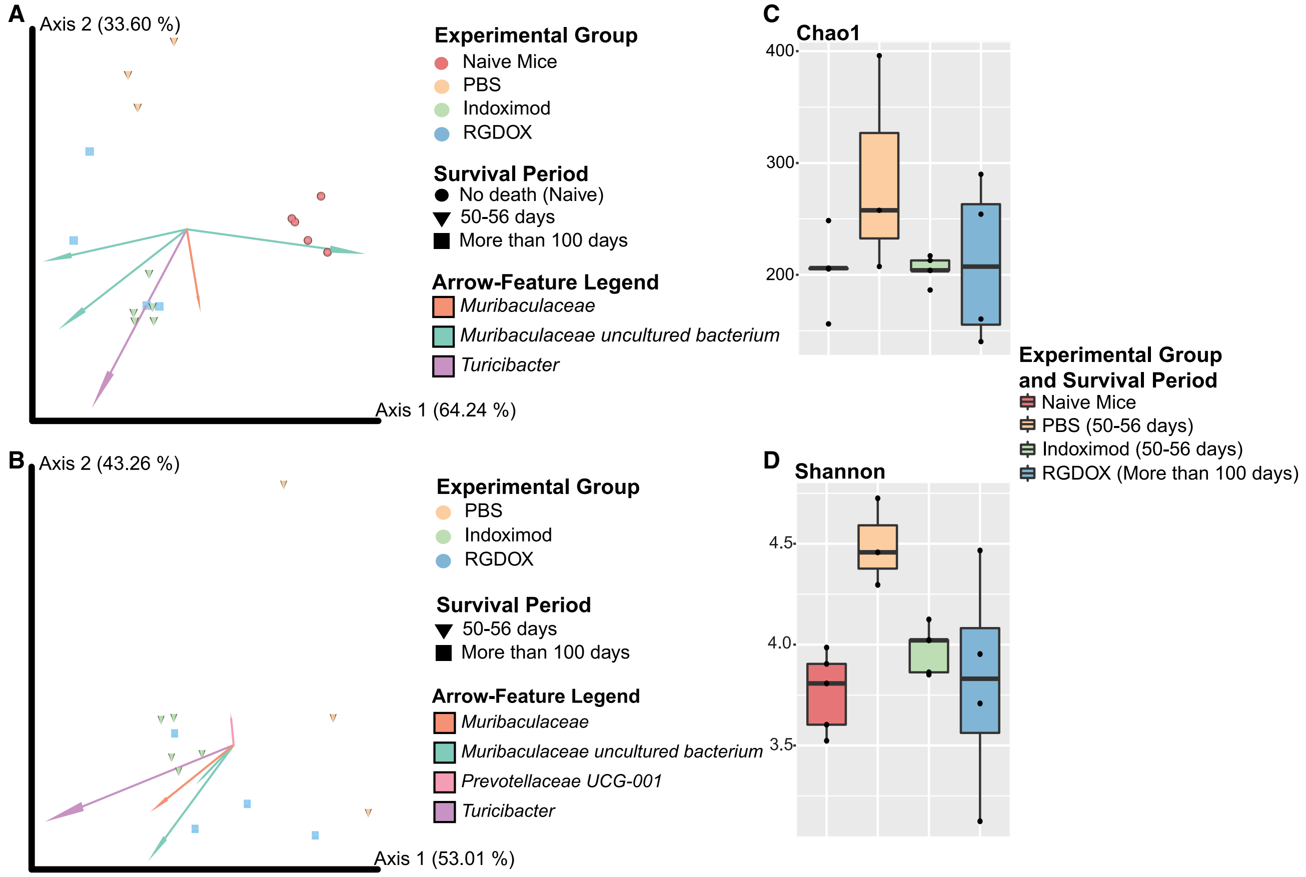

Figure 2. Combined overview of the microbiota changes showing that tumor presence alters gut bacterial structure and diversity in the GSC-005 mouse cohort

(A) We observe significant differences in the bacterial composition and structure (Aitchison distance) between groups: naive, PBS, indoximod, and Delta-24-RGDOX (PERMANOVA $p = 0.001$). The taxa arrow-features responsible for group clustering are highlighted in the compositional biplot. Each arrow corresponds to the specific feature (ASV), and its size indicates the strength of the relationship of that ASV with the community composition and grouping. (B) Differences in the bacterial structure with clear clustering between treatments (PERMANOVA $p = 0.004$), naive group excluded. (C) No differences in richness (Chao1) between groups (KW $p > 0.05$), although animals administered PBS have a tendency toward higher richness. (D) Shannon index and significant differences in the bacterial community diversity between naive vs. PBS with survival of 50–56 days (KW $p = 0.025347$) and PBS with survival of 50–56 days vs. indoximod with a survival of 50–56 days (KW $p = 0.025347$), observing a higher diversity in the GSC-005 glioma-bearing mice administered PBS. See also Table S4.

anti-inflammatory taxa, such as *Bifidobacterium* and *Akkermansia*, which could be associated with treatment response and correlated with long-term survival.

CD4$^+$ T cell depletion affects *Bifidobacterium* and other anti-inflammatory taxa important in viroimmunotherapy-treated mice

CD4$^+$ T cell-mediated adaptive immune responses are pivotal in maintaining intestinal homeostasis by distinguishing between commensal and pathogenic organisms.[33] Symbiotic commensals contribute to CD4$^+$ T cell differentiation,[33] and their depletion may affect the gut microbiota homeostasis.[34,35] We have previously reported that the combination of Delta-24-RGDOX and IDO inhibitors resulted in extended survival compared with those treatments administered as single agents.[24] Of interest, the activity of CD4$^+$ T cells was essential to achieve this anticancer effect since the depletion of these cells resulted in a survival similar to that of untreated mice. In this study, we further evaluate the importance CD4$^+$ helper T cells have in the modulation of the gut microbiota associated with the combination therapy of Delta-24-RGDOX and indoximod in intracranial GL261-5-bearing mice treated with depletion antibodies for CD4$^+$ T cells or an immunoglobulin G (IgG) isotype (Figure 1B). The median survival of the mice studied in this analysis was representative of our reported paper, showing a long-term survival (100 days) in mice treated with the combined therapy and similar survival between PBS-treated mice (43.00 days ±6.00) and those treated with the combined therapy and CD4$^+$ T cell depletion (53.0 days ±18.60) (Tables S2 and S3).[24] The analysis of bacterial community structure and composition showed significant differences between the treatment groups (PERMANOVA $p = 0.003$; Table S6). This was highlighted by strong

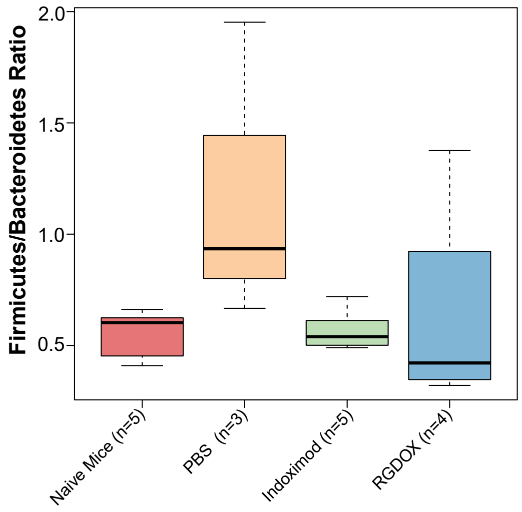

Figure 3. Boxplot depicting the F/B ratio for the experimental groups in the GSC-005 mouse cohort

This ratio is slightly reduced but not significantly (WRST $p > 0.05$; Table S5) in Delta-24-RGDOX-treated mice compared with all other groups. Animals administered PBS showed a very high F/B ratio.

dissimilarity between PBS-treated mice and those treated with both immunomodulatory therapies (PERMANOVA $p = 0.015$; Table S6), where *Bifidobacterium* was responsible for sample clustering of the combined therapy (Figure 5A).

While alpha diversity metrics showed no significant differences in richness or diversity between the treatment groups (KW $p > 0.05$; Table S6), mice treated with the combination therapy with $CD4^+$ T cell depletion tended to have lower richness compared with the treated mice with functional $CD4^+$ T cells and a higher richness than those bearing tumors and that were not treated (Figure 5B). Particularly, the F/B ratio, although not significantly reduced (WRST $p > 0.05$) in the immunomodulatory treated animals without $CD4^+$ T cell depletion, there seems to be a slight decrease in dysbiosis (Figure 5C; Table S6). In $CD4^+$ T cell-depleted mice treated with the combined therapy, we found a lower abundance of Actinobacteria (Figure 5D) corresponding at the genus level to a decrease of *Bifidobacterium* and *Lactobacillus* Figure 5E). These anti-inflammatory taxa were, however, increased in mice with functional $CD4^+$ T cells (Figures 5D and 5E).

The multivariable association model, MaAslin2, showed that $CD4^+$ T cell depletion in mice treated with the combined therapy resulted in a significant decrease in the phyla Actinobacteria (Figure 6A) and Verrucomicrobia (FDR $p = 3.073e-02$) (Figure 6B), highlighted by the genera *Bifidobacterium* (Figure 6C) and *Akkermansia* (FDR $p = 9.691e-02$) (Figure 6D), respectively. In addition, other anti-inflammatory taxa were found to be reduced in the $CD4^+$ T cell-depleted mice and included *Lactobacillus* (FDR $p = 1.093e-01$) (Figure 6E), Ruminococcaceae UCG 014 (FDR $p = 1.623e-01$) (Figure 6F), *Muribaculum* (FDR $p = 4.934e-03$) (Figure 6G), and Lachnospiraceae A2 (FDR $p = 1.623e-01$) (Figure 6H) (See also Figure S3). This analysis demonstrates that $CD4^+$ T cell depletion affects the levels of *Bifidobacterium* and other anti-inflammatory taxa that may be associated with efficacy of the combined therapy.

DISCUSSION

This is the first study describing the changes in murine gastrointestinal microbiota associated with oncolytic viral therapy efficacy against GBM. Although reversing tumor-mediated immunosuppression is one of the key elements necessary for immunotherapy to be successful, our results show that viroimmunotherapy (Delta-24-RGDOX)-treated animals had a higher survival compared with those treated with indoximod alone.[24] These IDO inhibitor-treated animals, although they had a median survival slightly higher than the controls, their biota had significant levels of *Turicibacter*, which were not detected in the naive and PBS-treated animals but that certainly had to be present in the community to increase its abundance in this group. This taxon, often associated with a healthy host, including probiotic properties, has been found in association with ICI response[36] and anti-inflammatory effects.[37]

Tumor injection affects the bacterial community structure and diversity by yet undescribed mechanisms. A skew toward a higher F/B ratio in the control mice of both cohorts shares similarities with that observed in obese individuals, indicating dysbiosis.[38,39] Changes in microbiota due to physiological alterations associated with the tumor might account for resistance to cancer therapies such as chemotherapy drugs[40] and immunotherapy.[41] Our results highlight the resemblance in gut diversity between viroimmunotherapy-treated mice and the naive group. Chemotherapies, such as irinotecan, affect the composition of the gut microbiome. Still, an important bidirectionality has been proven to occur, with the gut microbiota inducing enzymatic changes to the drug, changing its efficacy, and impacting gut homeostasis.[42,43] In this study, tumors seem to induce a change in the microbiota, with communities returning to a homeostatic state upon viroimmunotherapy—in fact, to a similar state as to how it was before tumor establishment.

Healthy naive animals (non-tumor bearing) displayed significant amounts of Muribaculaceae; members of this taxon are propionate producers and often found to be associated with gut health and mouse longevity.[44] We used two glioma cohorts to address the possibility of obtaining results due to the specific genetic makeup of a single model. In both models, we observed an enrichment of anti-inflammatory taxa associated with viroimmunotherapy treatment, supporting our hypothesis that gut bacterial signatures are associated with oncolytic viral therapy efficacy. Some of the most notorious taxonomic changes observed in the viroimmunotherapy-treated mice (GSC-005 GBM cohort) with prolonged survival included an increase in the phyla Actinobacteria and Verrucomicrobia and genera *Bifidobacterium* and *Akkermansia*, respectively. Other studies have confirmed the role of these genera in association with response improvement to ICIs.[27,28] In fact, mice undergoing anti-PD-L1 with *Bifidobacterium*

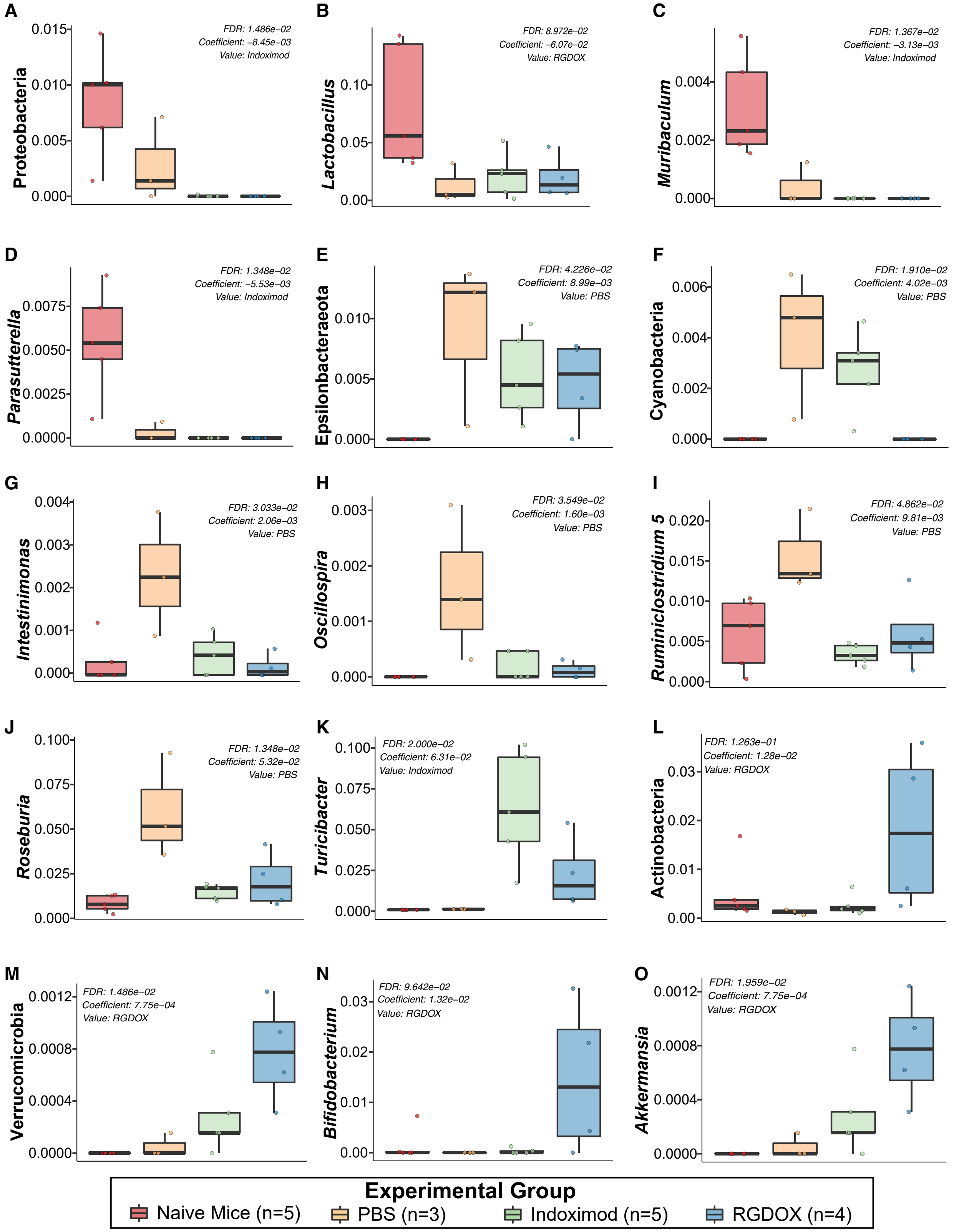

(legend on next page)

supplementation have demonstrated an improved antitumor T cell response through enhanced tumor control, stimulation of tumor-specific T cells, and increase in antigen-specific $CD8^+$ T cells within the tumor compared with non-*Bifidobacterium*-treated mice.[27] In another clinical study, patients with metastatic melanoma that responded to anti-PD-1 had a higher abundance of *Bifidobacterium longum*.[26] Inosine, a purine metabolite produced by *Bifidobacterium pseudolongum*, has been shown to enhance the efficacy of ICIs.[45] These studies support our hypothesis and confirm our results, pinpointing the potential role of certain gut microbes in influencing therapeutic efficacy. An important physiological function characteristic of the *Bifidobacterium* genus is the production of acetate and lactate through carbohydrate fermentation. These short-chain fatty acids (SCFAs) are further converted into butyrate by other commensal gut bacteria, such as *Akkermansia*.[46] Butyrate has been linked to the modulation of the $CD8^+$ T cell function in the tumor microenvironment and to antitumor properties.[47,48] Further studies have directly tested the anticancer attributes of *Bifidobacterium* against colorectal cancer,[49] highlighting the benefits of probiotic supplementation for enhancing patient response to therapies. The bidirectionality in the gut-glioma axis has been demonstrated in a study where GL261-5 GBM mice were administered antibiotics, and further evaluation showed that GBM growth was enhanced, and cytotoxic natural killer cells were reduced.[50] Depletion of $CD4^+$ T cells due to HIV infection has been shown to lead to dysbiosis of gut commensals[35] by affecting production of mucosal IgA.[34] This low-affinity IgA has been related to *Bifidobacterium* depletion.[51] In a previous study by our team, $CD4^+$ T cell depletion in a GBM mouse cohort resulted in lower mouse survival and lower efficacy of the oncolytic virus therapy,[24] here correlated with gut dysbiosis.

Similarly, previous studies have focused on the anti-inflammatory role of *Akkermansia muciniphila* in the colon[52] and response to PD-1 blockade.[53] An abundance of *Akkermansia* and *Bifidobacterium adolescentis* has been associated with better response to PD-1 blockade therapy and overall survival in non-small cell lung cancer patients.[53] A similar trend was observed in our data; mice treated with Delta-24-RGDOX had a higher overall survival compared with control or indoximod-treated animals with an increase in *Akkermansia* and *Bifidobacterium*. With data obtained from the GL261-5 GBM cohort, we found a reduction of anti-inflammatory taxa in Delta-24-RGDOX-treated mice that had $CD4^+$ helper T cell depletion. One detected taxon was within the Ruminococcaceae family which has also been associated with treatment efficacy.[30] It is true, however, that $CD4^+$ helper T cells may not only respond to the viroimmunotherapy but also to the commensal gut microbiota, which inherently poses some difficulty in interpreting fluctuations of bacterial taxa with T cell depletion. Nonetheless, our data showed that *Bifidobacterium* seemed to be a key player in viroimmunotherapy-treated mice that had $CD4^+$ helper T cells, as it was absent in animals with $CD4^+$ T cell depletion.

Our expanding knowledge of microbial taxonomy and its associated mechanisms sometimes challenges the notion of "good" versus "bad" microbes, and our study brings the opportunity for us to discuss these stereotypes. *Akkermansia*, and its species *A. muciniphila*, is a mucosal-dwelling anaerobic bacterium that is often found to be impacted (reduced) in a variety of metabolic disorders, including obesity.[54,55] It is generally a beneficial bacterium, and commercial probiotics have even been developed, as supplementation restores epithelial mucosal integrity in mouse models of diabetes and obesity.[56] However, these generalizations should be careful, given the duality we clearly find in our two small cohorts: an increase in *Akkermansia* in responders of the GSC-005 cohort and a reduction in the $CD4^+$ T cell-active cohort of GL261-5. In fact, studies of the gut microbiome of Parkinson's disease patients reported an increase in *Akkermansia*,[57] and in gnotobiotic mouse models with co-colonization of *Salmonella* and *Akkermansia*, there seems to be severe mucous degradation leading to leaky gut.[58] *Akkermansia* abundance has also been denoted to have a negative impact in Alzheimer's disease.[59] With this in mind, except for the universal knowledge of the importance of high levels of bifidobacteria, in the case of *Akkermansia*, the correct relative abundance is very important. Currently, what is accepted is that, depending on *Akkermansia* levels, there is a response resistance or improvement in immunotherapy.[53,60] An absence or overabundance of this taxon can lead to unresponsiveness to treatment, highlighting that adequate levels, depending on each case, would induce $CD4^+$ T helper cell activation as well as interferon (IFN)-related gene responses.[60] As of yet, there are no mechanistic studies that have really explored in detail these associations of *Akkermansia* with viroimmunotherapy, which is urgently needed.

Even with these interesting results, the study holds some limitations, which include a lack of appropriate controls in the GL261-5 GBM cohort and few biological replicates in some of the groups. Due to intrinsic gut bacterial changes between the two GBM cell lines used (GSC-005 and GL261-5), we were not able to use the single-agent controls from the GSC-005-implanted mice in our GL261-5 microbial cohort analysis or combine the replicates from the groups to maintain accuracy. Therefore, though these results are

Figure 4. The Actinobacteria and Verrucomicrobia phyla, highlighted by *Bifidobacterium* and *Akkermansia*, respectively, are important taxa in viroimmunotherapy mouse responders with longer survival in the GSC-005 cohort

The naive mice had higher prevalence of the phylum Proteobacteria (FDR p = 1.486e−02) (A) and the genera *Lactobacillus* (FDR p = 8.972e−02) (B), *Muribaculum* (FDR p = 1.367e−02) (C), and *Parasutterella* (FDR p = 1.348e−02) (D). The phyla Epsilonbacteraeota (FDR p = 4.266e−02) (E) and Cyanobacteria (FDR p = 1.910e−02) (F) are more characteristic of the glioma-bearing mice administered PBS, also reflected by higher levels of *Intestinimonas* (FDR p = 3.033e−02) (G), *Oscillospira* (FDR p = 3.549e−02) (H), *Ruminiclostridium 5* (FDR p = 4.862e−02) (I), and *Roseburia* (FDR p = 1.348e−02) (J). Indoximod-treated mice showed a significant increase only in *Turicibacter* (FDR p = 2.000e−02) (K). In mice treated with the oncolytic virus, we found an increase in Actinobacteria (FDR p = 1.263e−01) (L) and Verrucomicrobia (FDR p = 1.486e−02) (M). At the genus level, *Bifidobacterium* (FDR p = 9.642e−02) (N) and *Akkermansia* (FDR p = 1.959e−02) (O) are important biomarkers for Delta-24-RGDOX-treated mice.

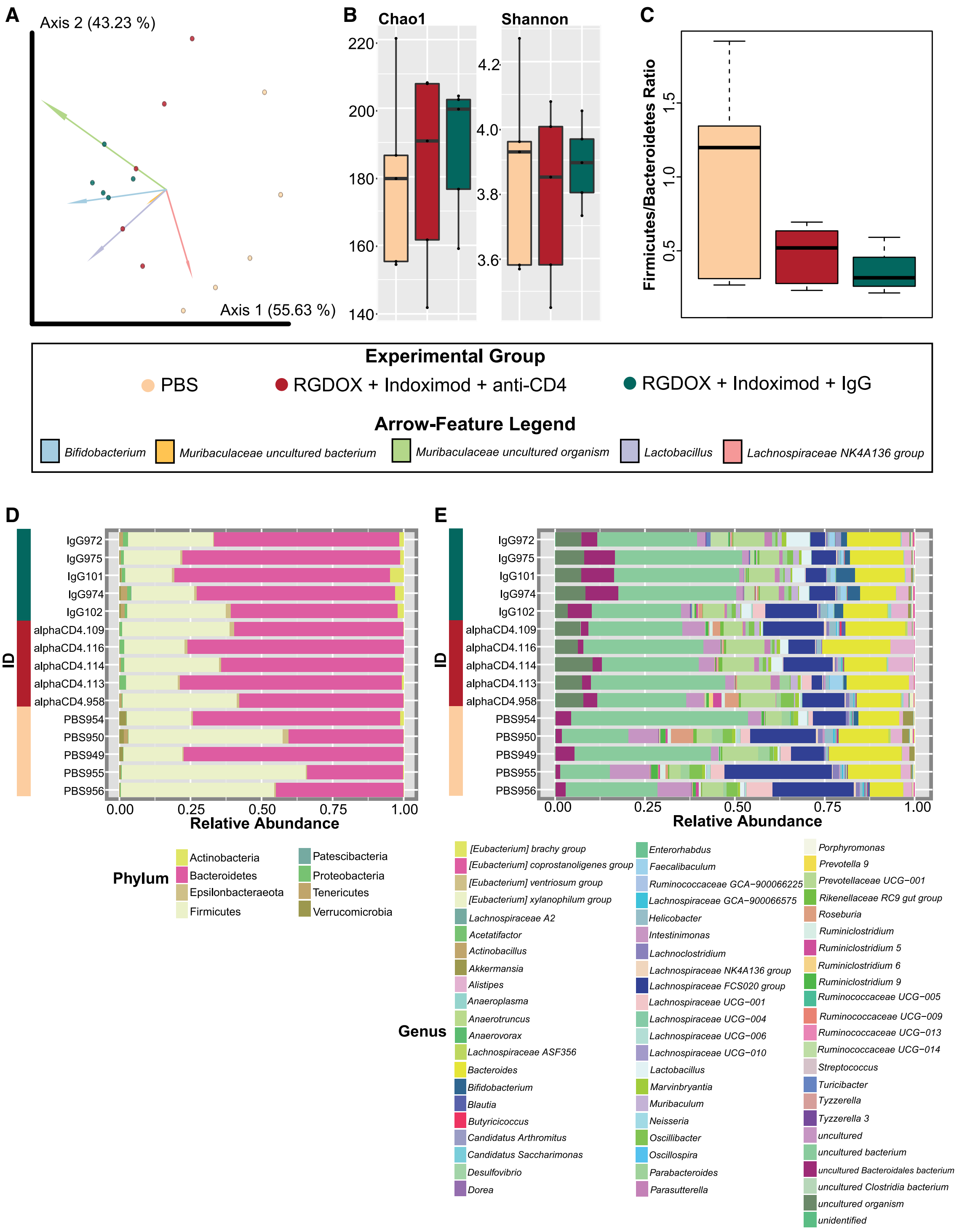

Figure 5. Combined overview of the microbiota shows that CD4$^+$ T cell depletion alters gut bacterial structure, diversity, and composition

(A) Significant differences in the bacterial composition and structure (Aitchison distance) between the groups: PBS, RGDOX + indoximod + anti-CD4, and RGDOX + indoximod + IgG (PERMANOVA p = 0.003). The features that strongly influence the clustering of the groups are highlighted in the compositional biplot, where *Bifidobacterium* is responsible for sample clustering of the RGDOX + indoximod + IgG group. (B) There are no differences in richness (Chao1 index) or diversity (Shannon index) between groups (KW p > 0.05), although animals treated with the oncolytic virus and indoximod with depleted CD4$^+$ T cells have a tendency toward lower richness and

(legend continued on next page)

very promising, a more robust analysis with appropriate controls is warranted.

These results highlight the intrinsic bidirectional communication that exists in the gut-glioma axis and the host immune system. *Bifidobacterium* was linked to a better antitumoral immune response and, consequently, to improvement of the therapeutic efficacy of the viroimmunotherapy. Nonetheless, more studies, including the mechanisms unfolded by the gut microbiota in modulating antitumor immunity, are necessary to determine a causal link between the gut microbiota and response to oncolytic viruses.

MATERIALS AND METHODS

Cell lines

Malignant gliomas are tumors characterized by high heterogeneity; therefore, our study used two intracranial glioma models, GL261-5 and GSC-005, to address the possibility of obtaining results due to the specific genetic makeup of a single model. The murine glioma cell line GL261-5 (a clone with slower *in vivo* growth kinetics compared with GL261, from the Tumor Bank Repository, National Cancer Institute, Frederick, MD, USA)[23] was cultured in Dulbecco's modified Eagle's medium with nutrient mixture F12 (DMEM/F12) (Corning). Additionally, the murine GSC-005 glioma cells (kindly provided by I.M. Verma, The Salk Institute for Biological Studies, CA, USA)[61] were maintained in DMEM/F12 supplemented with N2 (1×, Invitrogen), fibroblast growth factor 2 (20 ng/mL, PeproTech), epidermal growth factor (20 ng/mL, Promega), and heparin (50 μg/mL, Sigma). Cell cultures were further supplemented with 10% fetal bovine serum (HyCline Laboratories), penicillin (100 μg/mL, Corning), and streptomycin (100 μg/mL, Corning) and were stored under the following conditions: 37°C and 5% CO_2.

Oncolytic adenovirus Delta-24-RGDOX

Delta-24-RGDOX, a previously generated oncolytic adenovirus,[23] was amplified in human lung carcinoma A549 cells. Following the manufacturer's protocol for the Adenopure kit (Puresyn), the virions were collected and purified. To determine viral titers and replication of Delta-24-RGDOX, the plaque-forming units per milliliter were measured using conventional methods.

Indoximod, the IDO inhibitor

Indoximod (275 mg/kg, Sigma-Aldrich) 1-methyl-DL-tryptophan, was mixed in PBS and rotated overnight with 3-mm glass balls (Thomas Scientific, 3,000) to help resuspend the drug effectively.

In vivo studies

As reported previously for glioma implantation,[24] 5×10^4 GL261-5 or GSC-005 cells/mouse were implanted into the caudate nucleus of 7- to 10-week-old C57BL/6 mice using a guide-screw system as described previously.[62] On day 7, mice were randomly assigned to different experimental groups. For the GSC-005 GBM cohort, four experimental groups were determined: (1) PBS administered (n = 3), (2) indoximod treated (n = 5), and (3) Delta-24-RGDOX treated (n = 4); a naive group (n = 5) of mice with no tumor or treatment was added as a control. For the GL261-5 GBM cohort, mice were randomly divided into three experimental groups: (1) PBS plus IgG control antibody (Bio X Cell-InVivoMAb rat IgG2b isotype control, anti-keyhole limpet hemocyanin) (n = 5), (2) Delta-24-RGDOX plus indoximod and IgG, and (3) Delta-24-RGDOX plus indoximod and CD4-neutralizing antibody (Bio X Cell-InVivoMAb anti-mouse CD4 (clone GK1.5) (n = 5). Mice received intratumoral injections of Delta-24-RGDOX (5×10^7–1×10^8 plaque-forming units [PFUs]/dose) on days 7, 9, and 11, and indoximod (275 mg/kg) treatment was administered twice daily by oral gavage (5 days/week) beginning on day 7 and lasted until day 35 after tumor implantation. The neutralizing and control antibodies (200 μg/mouse) were administered every fourth day for nine rounds. All experimental protocols were approved by the University of Texas MD Anderson Cancer Center Institutional Animal Care and Use Committee and carried out according to the United States Department of Agriculture guidelines.

Mouse fecal pellet collection

Five mice were housed per treatment and separated into individual cages for fecal pellet collection. Sample collection was aseptically done using hot bead-sterilized forceps previously washed in alcohol between each animal to prevent cross-contamination. Fecal samples were collected on day 50 and day 37 after tumor implantation for the GSC-005 and GL261-5 GBM cohorts, respectively. Fecal pellets were stored in an ultra-low freezer (−80°C) until DNA extraction.

Genomic DNA extraction

gDNA was extracted from mice fecal pellets with the DNeasy PowerSoil Kit (QIAGEN, Germantown, MD, USA) with the following modifications of the manufacturer's protocol. (1) 200 μL of the bead solution was replaced with 200 μL of phenol:chloroform:isoamyl alcohol (PCI; pH 7–8). (2) C2 and C3 (100 μL of each) were mixed in one single step. (3) Equal parts of C4 and ethanol 100% were added to the supernatant. (4) Lysate was placed in a spin filter membrane using a QIAvac Vacuum System (QIAGEN). (5) Before adding solution C5, a wash of 650 μL of ethanol 100% was performed. (6) elution was made with 50 μL of warmed (55°C) C6 solution. gDNA quantifications were performed using the Qubit 1× dsDNA HS Assay Kit (high sensitivity; Thermo Fisher, Waltham, MA, USA) and the Qubit 2.0 fluorometer. The 16S rDNA gene hypervariable V4 region was amplified with the universal primers 515F (5′-GTGCCAGCMGCCGCGGTAA-3′) and 806R (5′-GGACTACHVGGGTWTCTAAT-3′) (https://earthmicrobiome.org/protocols-and-standards/). Amplicons were sequenced on Illumina MiSeq using a 2 × 250-bp paired-end protocol.

diversity compared with viroimmunotherapy-treated mice with functional $CD4^+$ T cells. (C) The F/B ratio for the experimental groups. This ratio is slightly reduced, but not significantly (WRST $p > 0.05$), in RGDOX/indoximod without $CD4^+$ T cell depletion mice compared with all other groups, whereas animals administered PBS showed a very high F/B ratio. (D) Taxonomic composition of gut bacteria at the phylum and genus levels between groups, where mice treated with combination therapies that had $CD4^+$ T cell depletion had a decreased abundance of Actinobacteria highlighted by lower levels of *Bifidobacterium* and *Lactobacillus*. See also Table S6.

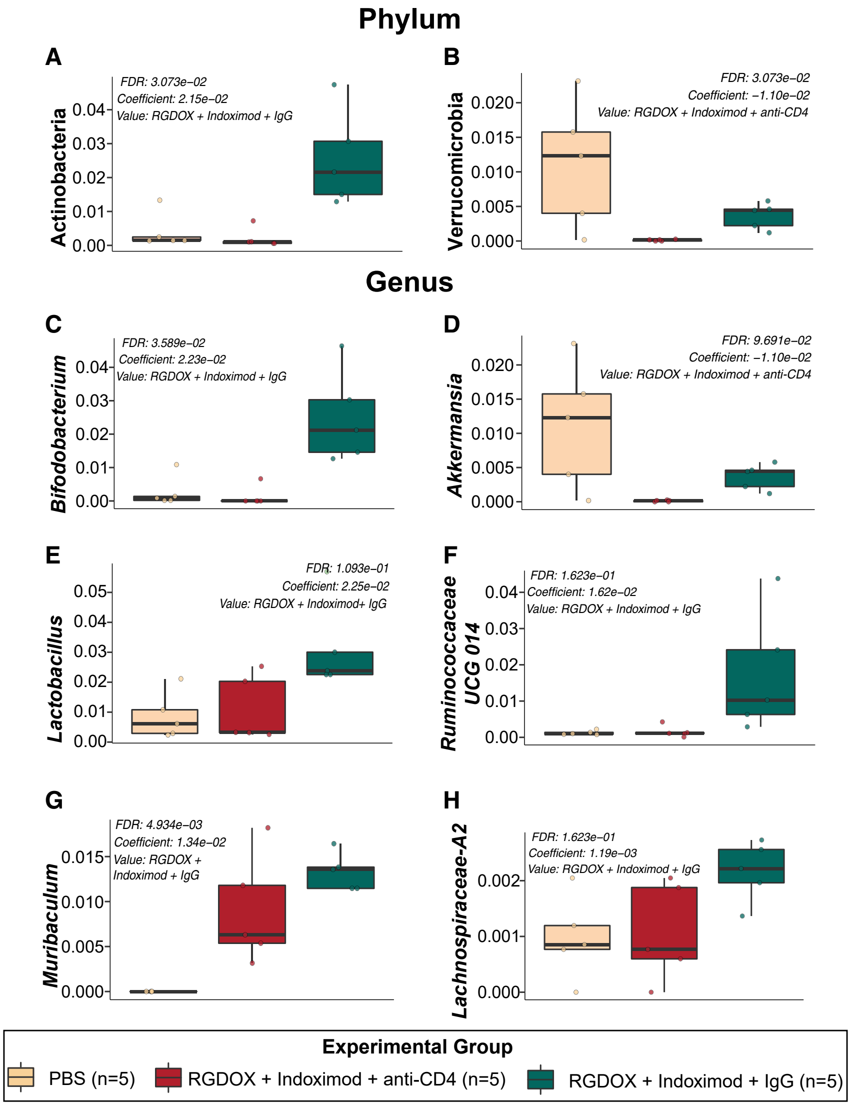

Figure 6. Significant taxonomic changes predicted by MaAsLin2 observed in viroimmunotherapy-treated mice with $CD4^{+}$ T cell depletion

The phyla Actinobacteria (FDR p = 3.073e−02) (A), characterized by the genus *Bifidobacterium* (C), and Verrucomicrobia (FDR p = 3.073e−02) (B), highlighted by the genus *Akkermansia* (FDR p = 9.691e−02) (D), were decreased in the gut microbiota of mice treated with the oncolytic virus and the IDO inhibitor when having depleted $CD4^{+}$ T cells. Other reduced biomarkers at the genus level of this group included *Lactobacillus* (FDR p = 1.093e−01) (E), Ruminococcaceae UCG 014 (FDR p = 1.623e−01) (F), *Muribaculum* (FDR p = 4.934e−03) (G), and Lachnospiraceae A2 (FDR p = 1.623e−01) (H).

The 16S rRNA demultiplexed amplicons were deposited and pre-processed for quality control in QIITA[63] and analyzed with the platforms QIIME2[64] and R (http://www.R-project.org/).

Quality control of sequencing reads

Raw 16S rDNA reads were pre-processed with a Phred offset of 30 and default split libraries (QIIMEq2 1.9.1). Trimming was settled at 250 bp, and a deblur workflow (deblur 2021.09)[65] was followed, with SILVA taxonomy[66,67] as a reference database with a minimum of 97% similarity threshold. The amplicon sequence variant (ASV) feature table was downloaded from QIITA, and elimination of singletons, mitochondria, and chloroplasts was performed. To continue downstream analyses on QIIME2,[64] the rarefaction level used was 6,448 reads per sample for the GSC-005 GBM cohort, 11,707 reads for the GL261-5 GBM cohort, and 8,835 for the control sample analysis for both cohorts.

Analyses of the microbial communities

Beta diversity

Compositional biplots were calculated with QIIME2[64] through DEICODE, plotting a variance-based diagram through a robust Aitchison principal-component analysis (PCA), which links specific features or taxa to a beta diversity ordination.[68] Each arrow corresponds to the specific feature (ASV) responsible for group clustering. Arrows respond to Euclidian distance from the origin, and their size indicates the strength of the relationship of that ASV to the community composition and grouping.[68,69] Since 16S rRNA sequencing provides a resolution of genus level, there are various similarly named ASVs that may have different functions.[68,69] The QIIME2 Emperor biplot selects these top feature arrows based on magnitude on all of the dimensions, while the scaling of the arrows is done by the largest value in each matrix.[68] PERMANOVA was employed to assess statistical significance using the QIIME2 pipeline.[64] For the control sample analysis of both cohorts (Figure S1), pairwise dissimilarities between communities were calculated by employing the Bray-Curtis distance and visualized through a non-metric multidimensional scaling (NMDS) with the phyloseq[70] and ggplot2[71] packages in R. ANOSIM tests were employed to assess statistical significance of group distances using the QIIME2 pipeline.

Alpha diversity and taxonomic profiles

We calculated Chao1 (richness) and Shannon index (diversity) alpha diversity metrics. In addition, taxonomic bar plots of phylum and genus levels were computed. Figures were built using the phyloseq,[70] vegan,[72] and ggplot2[71] packages in R. Non-parametric KW test was used to assess statistical significance for alpha diversity metrics.

F/B ratio

As Firmicutes and Bacteroidetes correspond to almost 90% of the gut microbiome, it is increasingly accepted that the F/B ratio could be

used as a biomarker in microbiota analyses.[73–75] Boxplots depicting the F/B ratio were built using the vegan package[72] in R. Statistical significance was addressed with WRST.

Microbial biomarkers

To detect bacterial biomarkers associated with the treatment efficiency of Delta-24-RGDOX against GBM with both cohorts, we selected the significant ASVs with a p value or q value of less than 0.05 to create phylum- and genus-level boxplots using the MaAslin2 library[76] in R. To perform the control sample analysis for both cohorts, we used a LEfSe (linear discriminant analysis effect size) algorithm[77] by employing a non-parametric KW rank-sum test on MicrobiomeAnalyst.[78,79] Taxa shown had a log linear discriminant analysis (LDA) score of 1.0 and a p value cutoff of 0.05. Last, for the GL261-5 GBM cohort, a selection of significant ASVs with a raw p value of less than 0.01 from a normalized biome with DESeq2[80] was visualized through a heatmap, specifically through the heatmap.2 function from the gplots package.[81]

DATA AND CODE AVAILABILITY

16S rRNA gene sequences can be found in the QIITA[63] study (sandbox ID 12724 [GL261-5 GBM cohort] and 12729 [GSC-005 GBM cohort]) (https://qiita.ucsd.edu/study/description/13433#). They are also available in the European Nucleotide Archive ENA Project (PRJEB58738, ERP143806).

SUPPLEMENTAL INFORMATION

Supplemental information can be found online at https://doi.org/10.1016/j.omton.2024.200787.

ACKNOWLEDGMENTS

This work was funded by the National Institutes of Health (NIH) UPR/MDACC: Partnership for Excellence in Cancer Research under award number 2U54CA096297-16 and 2U54CA096297-17 (to F.G.-V. and C.G.-M.); F31CA228207 (to T.T.N.), R01CA256006 (to J.F. and C.-G.M.), and P50CA127001 (to J.F.). Additional support to N.M.M.-V. was given by NIGMS-RISE Research Initiative for Scientific Enhancement award R25 GM061838. The work was partially supported by NIMHD-RCMI grant number U54 MD007600 and the Puerto Rico IDeA Networks of Biomedical Research Excellence, Advancing Competitive Biomedical Research in Puerto Rico, 5P20GM103475-20 (to F.G.-V.). This study also used MD Anderson's Research Animal Support Facility, which is supported in part by the NIH/NCI through MD Anderson's Cancer Center Support Grant P30CA01667.

AUTHOR CONTRIBUTIONS

Conceptualization, F.G.-V. and C.G.-M.; data curation, N.M.M.-V. and T.T.N.; formal analysis, N.M.M.V.; funding acquisition, F.G.-V. and C.G.-M.; investigation, N.M.M.-V. and T.T.N.; methodology, F.G.-V., C.G.-M., N.M.M.-V., T.T.N., and J.F.; project administration, F.G.-V. and C.G.-M.; resources, F.G.-V., C.G.-M., and J.F.; software, N.M.M.-V. and F.G.-V.; supervision, F.G.-V., C.G.-M., and J.F.; visualization, N.M.M.-V. and F.G.-V.; writing – original draft, N.M.M.-V. and F.G.-V.; writing – review & editing, N.M.M.-V., F.G.-V., C.G.-M., T.T.N., X.F., A.R.L.-R., and J.F.. All authors have read and agreed to the published version of the manuscript.

DECLARATION OF INTERESTS

C.G.-M. and J.F. have intellectual property related to this study.

REFERENCES

1. Ostrom, Q.T., Cioffi, G., Waite, K., Kruchko, C., and Barnholtz-Sloan, J.S. (2021). CBTRUS Statistical Report: Primary Brain and Other Central Nervous System Tumors Diagnosed in the United States in 2014-2018. Neuro Oncol. *23*, III1–III105. https://doi.org/10.1093/NEUONC/NOAB200.
2. Stupp, R., Hegi, M.E., Mason, W.P., van den Bent, M.J., Taphoorn, M.J.B., Janzer, R.C., Ludwin, S.K., Allgeier, A., Fisher, B., Belanger, K., et al. (2009). Effects of radiotherapy with concomitant and adjuvant temozolomide versus radiotherapy alone on survival in glioblastoma in a randomised phase III study: 5-year analysis of the EORTC-NCIC trial. Lancet Oncol. *10*, 459–466. https://doi.org/10.1016/S1470-2045(09)70025-7.
3. Ohka, F., Natsume, A., and Wakabayashi, T. (2012). Current Trends in Targeted Therapies for Glioblastoma Multiforme. Neurol. Res. Int. *2012*, 878425. https://doi.org/10.1155/2012/878425.
4. Wick, W., Puduvalli, V.K., Chamberlain, M.C., Van Den Bent, M.J., Carpentier, A.F., Cher, L.M., Mason, W., Weller, M., Hong, S., Musib, L., et al. (2010). Phase III Study of Enzastaurin Compared With Lomustine in the Treatment of Recurrent Intracranial Glioblastoma. J. Clin. Oncol. *28*, 1168–1174. https://doi.org/10.1200/JCO.2009.23.2595.
5. Larkin, J., Chiarion-Sileni, V., Gonzalez, R., Grob, J.-J., Rutkowski, P., Lao, C.D., Cowey, C.L., Schadendorf, D., Wagstaff, J., Dummer, R., et al. (2019). Five-Year Survival with Combined Nivolumab and Ipilimumab in Advanced Melanoma. N. Engl. J. Med. *381*, 1535–1546. https://doi.org/10.1056/NEJMOA1910836.
6. Wolchok, J.D., Chiarion-Sileni, V., Gonzalez, R., Rutkowski, P., Grob, J.-J., Cowey, C.L., Lao, C.D., Wagstaff, J., Schadendorf, D., Ferrucci, P.F., et al. (2017). Overall Survival with Combined Nivolumab and Ipilimumab in Advanced Melanoma. N. Engl. J. Med. *377*, 1345–1356. https://doi.org/10.1056/NEJMOA1709684.
7. Saha, D., Martuza, R.L., and Rabkin, S.D. (2017). Macrophage Polarization Contributes to Glioblastoma Eradication by Combination Immunovirotherapy and Immune Checkpoint Blockade. Cancer Cell *32*, 253–267.e5. https://doi.org/10.1016/J.CCELL.2017.07.006.
8. Fecci, P.E., Ochiai, H., Mitchell, D.A., Grossi, P.M., Sweeney, A.E., Archer, G.E., Cummings, T., Allison, J.P., Bigner, D.D., and Sampson, J.H. (2007). Systemic CTLA-4 blockade ameliorates glioma-induced changes to the CD4+ T cell compartment without affecting regulatory T-cell function. Clin. Cancer Res. *13*, 2158–2167. https://doi.org/10.1158/1078-0432.CCR-06-2070.
9. Park, J., Kim, C.G., Shim, J.K., Kim, J.H., Lee, H., Lee, J.E., Kim, M.H., Haam, K., Jung, I., Park, S.H., et al. (2019). Effect of combined anti-PD-1 and temozolomide therapy in glioblastoma. OncoImmunology *8*, e1525243. https://doi.org/10.1080/2162402X.2018.1525243.
10. Nayak, L., Molinaro, A.M., Peters, K., Clarke, J.L., Jordan, J.T., de Groot, J., Nghiemphu, L., Kaley, T., Colman, H., McCluskey, C., et al. (2021). Randomized Phase II and Biomarker Study of Pembrolizumab plus Bevacizumab versus Pembrolizumab Alone for Patients with Recurrent Glioblastoma. Clin. Cancer Res. *27*, 1048–1057. https://doi.org/10.1158/1078-0432.CCR-20-2500.
11. Reardon, D.A., Brandes, A.A., Omuro, A., Mulholland, P., Lim, M., Wick, A., Baehring, J., Ahluwalia, M.S., Roth, P., Bähr, O., et al. (2020). Effect of Nivolumab vs Bevacizumab in Patients With Recurrent Glioblastoma: The CheckMate 143 Phase 3 Randomized Clinical Trial. JAMA Oncol. *6*, 1003–1010. https://doi.org/10.1001/JAMAONCOL.2020.1024.
12. Weenink, B., French, P.J., Smitt, P.A.E.S., Debets, R., and Geurts, M. (2020). Immunotherapy in Glioblastoma: Current Shortcomings and Future Perspectives. Cancers *12*. https://doi.org/10.3390/CANCERS12030751.

13. Carpenter, A.B., Carpenter, A.M., Aiken, R., and Hanft, S. (2021). Oncolytic virus in gliomas: a review of human clinical investigations. Ann. Oncol. *32*, 968–982. https://doi.org/10.1016/j.annonc.2021.03.197.

14. Jiang, H., Gomez-Manzano, C., Aoki, H., Alonso, M.M., Kondo, S., McCormick, F., Xu, J., Kondo, Y., Bekele, B.N., Colman, H., et al. (2007). Examination of the therapeutic potential of Delta-24-RGD in brain tumor stem cells: role of autophagic cell death. J. Natl. Cancer Inst. *99*, 1410–1414. https://doi.org/10.1093/JNCI/DJM102.

15. Jiang, H., White, E.J., Ríos-Vicil, C.I., Xu, J., Gomez-Manzano, C., and Fueyo, J. (2011). Human Adenovirus Type 5 Induces Cell Lysis through Autophagy and Autophagy-Triggered Caspase Activity. J. Virol. *85*, 4720–4729. https://doi.org/10.1128/JVI.02032-10.

16. Fueyo, J., Alemany, R., Gomez-Manzano, C., Fuller, G.N., Khan, A., Conrad, C.A., Liu, T.J., Jiang, H., Lemoine, M.G., Suzuki, K., et al. (2003). Preclinical characterization of the antiglioma activity of a tropism-enhanced adenovirus targeted to the retinoblastoma pathway. J. Natl. Cancer Inst. *95*, 652–660. https://doi.org/10.1093/JNCI/95.9.652.

17. Jiang, H., Gomez-Manzano, C., Rivera-Molina, Y., Lang, F.F., Conrad, C.A., and Fueyo, J. (2015). Oncolytic adenovirus research evolution: from cell-cycle checkpoints to immune checkpoints. Curr. Opin. Virol. *13*, 33–39. https://doi.org/10.1016/J.COVIRO.2015.03.009.

18. Jiang, H., Clise-Dwyer, K., Ruisaard, K.E., Fan, X., Tian, W., Gumin, J., Lamfers, M.L., Kleijn, A., Lang, F.F., Yung, W.K.A., et al. (2014). Delta-24-RGD oncolytic adenovirus elicits anti-glioma immunity in an immunocompetent mouse model. PLoS One *9*, e97407. https://doi.org/10.1371/journal.pone.0097407.

19. Fueyo, J., Gomez-Manzano, C., Alemany, R., Lee, P.S., McDonnell, T.J., Mitlianga, P., Shi, Y.X., Levin, V.A., Yung, W.K., and Kyritsis, A.P. (2000). A mutant oncolytic adenovirus targeting the Rb pathway produces anti-glioma effect in vivo. Oncogene *19*, 2–12. https://doi.org/10.1038/SJ.ONC.1203251.

20. Lang, F.F., Conrad, C., Gomez-Manzano, C., Yung, W.K.A., Sawaya, R., Weinberg, J.S., Prabhu, S.S., Rao, G., Fuller, G.N., Aldape, K.D., et al. (2018). Phase I Study of DNX-2401 (Delta-24-RGD) Oncolytic Adenovirus: Replication and Immunotherapeutic Effects in Recurrent Malignant Glioma. J. Clin. Oncol. *36*, 1419–1427. https://doi.org/10.1200/JCO.2017.75.8219.

21. Nassiri, F., Patil, V., Yefet, L.S., Singh, O., Liu, J., Dang, R.M.A., Yamaguchi, T.N., Daras, M., Cloughesy, T.F., Colman, H., et al. (2023). Oncolytic DNX-2401 virotherapy plus pembrolizumab in recurrent glioblastoma: a phase 1/2 trial. Nat. Med. *29*, 1370–1378. https://doi.org/10.1038/s41591-023-02347-y.

22. Gállego Pérez-Larraya, J., Garcia-Moure, M., Labiano, S., Patiño-García, A., Dobbs, J., Gonzalez-Huarriz, M., Zalacain, M., Marrodan, L., Martinez-Velez, N., Puigdelloses, M., et al. (2022). Oncolytic DNX-2401 Virus for Pediatric Diffuse Intrinsic Pontine Glioma. N. Engl. J. Med. *386*. https://doi.org/10.1056/nejmoa2202028.

23. Jiang, H., Rivera-Molina, Y., Gomez-Manzano, C., Clise-Dwyer, K., Bover, L., Vence, L.M., Yuan, Y., Lang, F.F., Toniatti, C., Hossain, M.B., and Fueyo, J. (2017). Oncolytic adenovirus and tumor-targeting immune modulatory therapy improve autologous cancer vaccination. Cancer Res. *77*, 3894–3907. https://doi.org/10.1158/0008-5472.CAN-17-0468.

24. Nguyen, T.T., Shin, D.H., Sohoni, S., Singh, S.K., Rivera-Molina, Y., Jiang, H., Fan, X., Gumin, J., Lang, F.F., Alvarez-Breckenridge, C., et al. (2022). Reshaping the tumor microenvironment with oncolytic viruses, positive regulation of the immune synapse, and blockade of the immunosuppressive oncometabolic circuitry. J. Immunother. Cancer *10*, e004935. https://doi.org/10.1136/JITC-2022-004935.

25. Hanahan, D. (2022). Hallmarks of Cancer: New Dimensions. Cancer Discov. *12*, 31–46. https://doi.org/10.1158/2159-8290.CD-21-1059.

26. Matson, V., Fessler, J., Bao, R., Chongsuwat, T., Zha, Y., Alegre, M.L., Luke, J.J., and Gajewski, T.F. (2018). The commensal microbiome is associated with anti-PD-1 efficacy in metastatic melanoma patients. Science (New York, N.Y.) *359*, 104–108. https://doi.org/10.1126/SCIENCE.AAO3290.

27. Sivan, A., Corrales, L., Hubert, N., Williams, J.B., Aquino-Michaels, K., Earley, Z.M., Benyamin, F.W., Lei, Y.M., Jabri, B., Alegre, M.L., et al. (2015). Commensal Bifidobacterium promotes antitumor immunity and facilitates anti–PD-L1 efficacy. Science (New York, N.Y.) *350*, 1084–1089. https://doi.org/10.1126/SCIENCE.AAC4255.

28. Routy, B., Le Chatelier, E., Derosa, L., Duong, C.P.M., Alou, M.T., Daillère, R., Fluckiger, A., Messaoudene, M., Rauber, C., Roberti, M.P., et al. (2018). Gut microbiome influences efficacy of PD-1-based immunotherapy against epithelial tumors. Science (New York, N.Y.) *359*, 91–97. https://doi.org/10.1126/SCIENCE.AAN3706.

29. Vétizou, M., Pitt, J.M., Daillère, R., Lepage, P., Waldschmitt, N., Flament, C., Rusakiewicz, S., Routy, B., Roberti, M.P., Duong, C.P.M., et al. (2015). Anticancer immunotherapy by CTLA-4 blockade relies on the gut microbiota. Science (New York, N.Y.) *350*, 1079–1084. https://doi.org/10.1126/SCIENCE.AAD1329.

30. Gopalakrishnan, V., Spencer, C.N., Nezi, L., Reuben, A., Andrews, M.C., Karpinets, T.V., Prieto, P.A., Vicente, D., Hoffman, K., Wei, S.C., et al. (2018). Gut microbiome modulates response to anti–PD-1 immunotherapy in melanoma patients. Science (New York, N.Y.) *359*, 97–103. https://doi.org/10.1126/SCIENCE.AAN4236.

31. Stojanov, S., Berlec, A., and Štrukelj, B. (2020). The influence of probiotics on the firmicutes/bacteroidetes ratio in the treatment of obesity and inflammatory bowel disease. Microorganisms *8*, 1715. https://doi.org/10.3390/microorganisms8111715.

32. Lynch, J.B., Gonzalez, E.L., Choy, K., Faull, K.F., Jewell, T., Arellano, A., Liang, J., Yu, K.B., Paramo, J., and Hsiao, E.Y. (2023). Gut microbiota Turicibacter strains differentially modify bile acids and host lipids. Nat. Commun. *14*, 3669. https://doi.org/10.1038/s41467-023-39403-7.

33. Sorini, C., Cardoso, R.F., Gagliani, N., and Villablanca, E.J. (2018). Commensal Bacteria-Specific CD4+ T Cell Responses in Health and Disease. Front. Immunol. *9*, 2667. https://doi.org/10.3389/FIMMU.2018.02667.

34. Onabajo, O.O., and Mattapallil, J.J. (2021). Gut Microbiome Homeostasis and the CD4 T- Follicular Helper Cell IgA Axis in Human Immunodeficiency Virus Infection. Front. Immunol. *12*, 657679. https://doi.org/10.3389/FIMMU.2021.657679.

35. Lu, D., Zhang, J.B., Wang, Y.X., Geng, S.T., Zhang, Z., Xu, Y., Li, S.Y., Wang, K.H., and Kuang, Y.Q. (2021). Association between CD4+ T cell counts and gut microbiota and serum cytokines levels in HIV-infected immunological non-responders. BMC Infect. Dis. *21*, 742. https://doi.org/10.1186/S12879-021-06491-Z.

36. Hamada, K., Isobe, J., Hattori, K., Hosonuma, M., Baba, Y., Murayama, M., Narikawa, Y., Toyoda, H., Funayama, E., Tajima, K., et al. (2023). Turicibacter and Acidaminococcus predict immune-related adverse events and efficacy of immune checkpoint inhibitor. Front. Immunol. *14*, 1164724. https://doi.org/10.3389/fimmu.2023.1164724.

37. Liu, W., Crott, J.W., Lyu, L., Pfalzer, A.C., Li, J., Choi, S.W., Yang, Y., Mason, J.B., and Liu, Z. (2016). Diet-and genetically-induced obesity produces alterations in the microbiome, inflammation and Wnt pathway in the intestine of Apc+/1638N mice: Comparisons and contrasts. J. Cancer *7*, 1780–1790. https://doi.org/10.7150/jca.15792.

38. Koliada, A., Syzenko, G., Moseiko, V., Budovska, L., Puchkov, K., Perederiy, V., Gavalko, Y., Dorofeyev, A., Romanenko, M., Tkach, S., et al. (2017). Association between body mass index and Firmicutes/Bacteroidetes ratio in an adult Ukrainian population. BMC Microbiol. *17*, 120–126. https://doi.org/10.1186/s12866-017-1027-1.

39. Palmas, V., Pisanu, S., Madau, V., Casula, E., Deledda, A., Cusano, R., Uva, P., Vascellari, S., Loviselli, A., Manzin, A., and Velluzzi, F. (2021). Gut microbiota markers associated with obesity and overweight in Italian adults. Sci. Rep. *11*, 5532–5614. https://doi.org/10.1038/s41598-021-84928-w.

40. Viaud, S., Saccheri, F., Mignot, G., Yamazaki, T., Daillère, R., Hannani, D., Enot, D.P., Pfirschke, C., Engblom, C., Pittet, M.J., et al. (2013). The intestinal microbiota modulates the anticancer immune effects of cyclophosphamide. Science (New York, N.Y.) *342*, 971–976. https://doi.org/10.1126/SCIENCE.1240537.

41. Iida, N., Dzutsev, A., Stewart, C.A., Smith, L., Bouladoux, N., Weingarten, R.A., Molina, D.A., Salcedo, R., Back, T., Cramer, S., et al. (2013). Commensal Bacteria Control Cancer Response to Therapy by Modulating the Tumor Microenvironment. Science (New York, N.Y.) *342*, 967–970. https://doi.org/10.1126/SCIENCE.1240527.

42. Bhatt, A.P., Pellock, S.J., Biernat, K.A., Walton, W.G., Wallace, B.D., Creekmore, B.C., Letertre, M.M., Swann, J.R., Wilson, I.D., Roques, J.R., et al. (2020). Targeted inhibition of gut bacterial β-glucuronidase activity enhances anticancer drug efficacy. Proc. Natl. Acad. Sci. USA *117*, 7374–7381. https://doi.org/10.1073/pnas.1918095117.

43. Chamseddine, A.N., Ducreux, M., Armand, J.P., Paoletti, X., Satar, T., Paci, A., and Mir, O. (2019). Intestinal bacterial β -glucuronidase as a possible predictive biomarker of irinotecan-induced diarrhea severity. Pharmacol. Ther. *199*, 1–15. https://doi.org/10.1016/j.pharmthera.2019.03.002.

44. Smith, B.J., Miller, R.A., and Schmidt, T.M. (2021). Muribaculaceae Genomes Assembled from Metagenomes Suggest Genetic Drivers of Differential Response to Acarbose Treatment in Mice. mSphere *6*, e0085121. https://doi.org/10.1128/MSPHERE.00851-21.

45. Mager, L.F., Burkhard, R., Pett, N., Cooke, N.C.A., Brown, K., Ramay, H., Paik, S., Stagg, J., Groves, R.A., Gallo, M., et al. (2020). Microbiome-derived inosine modulates response to checkpoint inhibitor immunotherapy. Science *369*, 1481–1489. https://doi.org/10.1126/science.abc3421.

46. Rivière, A., Selak, M., Lantin, D., Leroy, F., and De Vuyst, L. (2016). Bifidobacteria and Butyrate-Producing Colon Bacteria: Importance and Strategies for Their Stimulation in the Human Gut. Front. Microbiol. *7*, 979. https://doi.org/10.3389/FMICB.2016.00979.

47. He, Y., Fu, L., Li, Y., Wang, W., Gong, M., Zhang, J., Dong, X., Huang, J., Wang, Q., Mackay, C.R., et al. (2021). Gut microbial metabolites facilitate anticancer therapy efficacy by modulating cytotoxic CD8+ T cell immunity. Cell Metab. *33*, 988–1000.e7. https://doi.org/10.1016/J.CMET.2021.03.002.

48. Wei, H., Chen, L., Lian, G., Yang, J., Li, F., Zou, Y., Lu, F., and Yin, Y. (2018). Antitumor mechanisms of bifidobacteria. Oncol. Lett. *16*, 3–8. https://doi.org/10.3892/OL.2018.8692.

49. Parisa, A., Roya, G., Mahdi, R., Shabnam, R., Maryam, E., and Malihe, T. (2020). Anti-cancer effects of Bifidobacterium species in colon cancer cells and a mouse model of carcinogenesis. PLoS One *15*. https://doi.org/10.1371/JOURNAL.PONE.0232930.

50. D'Alessandro, G., Antonangeli, F., Marrocco, F., Porzia, A., Lauro, C., Santoni, A., and Limatola, C. (2020). Gut microbiota alterations affect glioma growth and innate immune cells involved in tumor immunosurveillance in mice. Eur. J. Immunol. *50*, 705–711. https://doi.org/10.1002/EJI.201948354.

51. Kawamoto, S., Tran, T.H., Maruya, M., Suzuki, K., Doi, Y., Tsutsui, Y., Kato, L.M., and Fagarasan, S. (2012). The inhibitory receptor PD-1 regulates IgA selection and bacterial composition in the gut. Science *336*, 485–489. https://doi.org/10.1126/science.1217718.

52. Raftar, S.K.A., Ashrafian, F., Abdollahiyan, S., Yadegar, A., Moradi, H.R., Masoumi, M., Vaziri, F., Moshiri, A., Siadat, S.D., and Zali, M.R. (2022). The anti-inflammatory effects of Akkermansia muciniphila and its derivates in HFD/CCL4-induced murine model of liver injury. Sci. Rep. *12*, 2453–2514. https://doi.org/10.1038/s41598-022-06414-1.

53. Derosa, L., Routy, B., Thomas, A.M., Iebba, V., Zalcman, G., Friard, S., Mazieres, J., Audigier-Valette, C., Moro-Sibilot, D., Goldwasser, F., et al. (2022). Intestinal Akkermansia muciniphila predicts clinical response to PD-1 blockade in patients with advanced non-small-cell lung cancer. Nat. Med. *28*, 315–324. https://doi.org/10.1038/S41591-021-01655-5.

54. Zhou, Q., Zhang, Y., Wang, X., Yang, R., Zhu, X., Zhang, Y., Chen, C., Yuan, H., Yang, Z., and Sun, L. (2020). Gut bacteria Akkermansia is associated with reduced risk of obesity: evidence from the American Gut Project. Nutr. Metab. *17*, 90. https://doi.org/10.1186/s12986-020-00516-1.

55. Depommier, C., Everard, A., Druart, C., Plovier, H., Van Hul, M., Vieira-Silva, S., Falony, G., Raes, J., Maiter, D., Delzenne, N.M., et al. (2019). Supplementation with Akkermansia muciniphila in overweight and obese human volunteers: a proof-of-concept exploratory study. Nat. Med. *25*, 1096–1103. https://doi.org/10.1038/s41591-019-0495-2.

56. Plovier, H., Everard, A., Druart, C., Depommier, C., Van Hul, M., Geurts, L., Chilloux, J., Ottman, N., Duparc, T., Lichtenstein, L., et al. (2017). A purified membrane protein from Akkermansia muciniphila or the pasteurized bacterium improves metabolism in obese and diabetic mice. Nat. Med. *23*, 107–113. https://doi.org/10.1038/nm.4236.

57. Heintz-Buschart, A., Pandey, U., Wicke, T., Sixel-Döring, F., Janzen, A., Sittig-Wiegand, E., Trenkwalder, C., Oertel, W.H., Mollenhauer, B., and Wilmes, P. (2018). The nasal and gut microbiome in Parkinson's disease and idiopathic rapid eye movement sleep behavior disorder. Mov. Disord. *33*, 88–98. https://doi.org/10.1002/mds.27105.

58. Gorecki, A.M., Preskey, L., Bakeberg, M.C., Kenna, J.E., Gildenhuys, C., MacDougall, G., Dunlop, S.A., Mastaglia, F.L., Akkari, P.A., Koengten, F., and Anderton, R.S. (2019). Altered gut microbiome in Parkinson's disease and the influence of lipopolysaccharide in a human α-synuclein over-expressing mouse model. Front. Neurosci. *13*, 839. https://doi.org/10.3389/fnins.2019.00839.

59. Vogt, N.M., Kerby, R.L., Dill-McFarland, K.A., Harding, S.J., Merluzzi, A.P., Johnson, S.C., Carlsson, C.M., Asthana, S., Zetterberg, H., Blennow, K., et al. (2017). Gut microbiome alterations in Alzheimer's disease. Sci. Rep. *7*, 13537. https://doi.org/10.1038/s41598-017-13601-y.

60. Li, L., and McAllister, F. (2022). Too much water drowned the miller: Akkermansia determines immunotherapy responses Cell Reports Medicine. Cell Rep. Med. *3*, 100642. https://doi.org/10.1016/j.xcrm.2022.100642.

61. Marumoto, T., Tashiro, A., Friedmann-Morvinski, D., Scadeng, M., Soda, Y., Gage, F.H., and Verma, I.M. (2009). Development of a novel mouse glioma model using lentiviral vectors. Nat. Med. *15*, 110–116. https://doi.org/10.1038/NM.1863.

62. Lal, S., Lacroix, M., Tofilon, P., Fuller, G.N., Sawaya, R., and Lang, F.F. (2000). An implantable guide-screw system for brain tumor studies in small animals. J. Neurosurg. *92*, 326–333. https://doi.org/10.3171/JNS.2000.92.2.0326.

63. Gonzalez, A., Navas-Molina, J.A., Kosciolek, T., McDonald, D., Vázquez-Baeza, Y., Ackermann, G., DeReus, J., Janssen, S., Swafford, A.D., Orchanian, S.B., et al. (2018). Qiita: rapid, web-enabled microbiome meta-analysis. Nat. Methods *15*, 796–798. https://doi.org/10.1038/S41592-018-0141-9.

64. Bolyen, E., Rideout, J.R., Dillon, M.R., Bokulich, N.A., Abnet, C.C., Al-Ghalith, G.A., Alexander, H., Alm, E.J., Arumugam, M., Asnicar, F., et al. (2019). Reproducible, interactive, scalable and extensible microbiome data science using QIIME 2. Nat. Biotechnol. *37*, 852–857. https://doi.org/10.1038/S41587-019-0209-9.

65. Amir, A., McDonald, D., Navas-Molina, J.A., Kopylova, E., Morton, J.T., Zech Xu, Z., Kightley, E.P., Thompson, L.R., Hyde, E.R., Gonzalez, A., and Knight, R. (2017). Deblur Rapidly Resolves Single-Nucleotide Community Sequence Patterns. mSystems *2*, e00191. https://doi.org/10.1128/MSYSTEMS.00191-16.

66. Quast, C., Pruesse, E., Yilmaz, P., Gerken, J., Schweer, T., Yarza, P., Peplies, J., and Glöckner, F.O. (2013). The SILVA ribosomal RNA gene database project: improved data processing and web-based tools. Nucleic Acids Res. *41*, D590–D596. Database issue). https://doi.org/10.1093/NAR/GKS1219.

67. Pruesse, E., Quast, C., Knittel, K., Fuchs, B.M., Ludwig, W., Peplies, J., and Glöckner, F.O. (2007). SILVA: a comprehensive online resource for quality checked and aligned ribosomal RNA sequence data compatible with ARB. Nucleic Acids Res. *35*, 7188–7196. https://doi.org/10.1093/NAR/GKM864.

68. Martino, C., Morton, J.T., Marotz, C.A., Thompson, L.R., Tripathi, A., Knight, R., and Zengler, K. (2019). A Novel Sparse Compositional Technique Reveals Microbial Perturbations. mSystems *4*. e00191-16. https://doi.org/10.1128/msystems.00016-19.

69. Liu, B., Sträuber, H., Centler, F., Harms, H., da Rocha, U.N., and Kleinsteuber, S. (2023). Functional Redundancy Secures Resilience of Chain Elongation Communities upon pH Shifts in Closed Bioreactor Ecosystems. Environ. Sci. Technol. *57*, 18350–18361. https://doi.org/10.1021/acs.est.2c09573.

70. McMurdie, P.J., and Holmes, S. (2013). Phyloseq: An R Package for Reproducible Interactive Analysis and Graphics of Microbiome Census Data. PLoS One *8*, e61217. https://doi.org/10.1371/journal.pone.0061217.

71. Wickham, H. (2009). Ggplot2: Elegant Graphics for Data Analysis, 2nd ed. (Springer). https://doi.org/10.1007/978-0-387-98141-3.

72. Oksanen, J., Guillaume Blanchet, F., Friendly, M., Kindt, R., Legendre, P., McGlinn, D., Minchin, P.R., O'Hara, R.B., Simpson, G.L., Solymos, P., et al. (2019). Vegan: Community Ecology Package.

73. Bahar-Tokman, H., Demirci, M., Keskin, F.E., Cagatay, P., Taner, Z., Ozturk-Bakar, Y., Ozyazar, M., Kiraz, N., and Kocazeybek, B.S. (2022). Firmicutes/Bacteroidetes Ratio in the Gut Microbiota and IL-1β, IL-6, IL-8, TLR2, TLR4, TLR5 Gene Expressions in Type 2 Diabetes. Clin. Lab. *68*. https://doi.org/10.7754/Clin.Lab.2022.211244.

74. Petakh, P., Oksenych, V., and Kamyshnyi, A. (2023). The F/B ratio as a biomarker for inflammation in COVID-19 and T2D: Impact of metformin. Biomed. Pharmacother. *163*, 114892. https://doi.org/10.1016/j.biopha.2023.114892.

75. An, J., Kwon, H., and Kim, Y.J. (2023). The Firmicutes/Bacteroidetes Ratio as a Risk Factor of Breast Cancer. J. Clin. Med. *12*, 2216. https://doi.org/10.3390/jcm12062216.

76. Mallick, H., Rahnavard, A., McIver, L.J., Ma, S., Zhang, Y., Nguyen, L.H., Tickle, T.L., Weingart, G., Ren, B., Schwager, E.H., et al. (2021). Multivariable association discovery in population-scale meta-omics studies. PLoS Comput. Biol. *17*, e1009442. https://doi.org/10.1371/journal.pcbi.1009442.

77. Segata, N., Izard, J., Waldron, L., Gevers, D., Miropolsky, L., Garrett, W.S., and Huttenhower, C. (2011). Metagenomic biomarker discovery and explanation. Genome Biol. *12*, R60. https://doi.org/10.1186/GB-2011-12-6-R60.

78. Chong, J., Liu, P., Zhou, G., and Xia, J. (2020). Using MicrobiomeAnalyst for comprehensive statistical, functional, and meta-analysis of microbiome data. Nat. Protoc. *15*, 799–821. https://doi.org/10.1038/S41596-019-0264-1.

79. Dhariwal, A., Chong, J., Habib, S., King, I.L., Agellon, L.B., and Xia, J. (2017). MicrobiomeAnalyst: a web-based tool for comprehensive statistical, visual and meta-analysis of microbiome data. Nucleic Acids Res. *45*, W180–W188. https://doi.org/10.1093/NAR/GKX295.

80. Love, M.I., Huber, W., and Anders, S. (2014). Moderated estimation of fold change and dispersion for RNA-seq data with DESeq2. Genome Biol. *15*, 550. https://doi.org/10.1186/S13059-014-0550-8.

81. Warnes, G.R., Bolker, B., Bonebakker, L., Gentleman, R., Huber, W., Liaw, A., Lumley, T., Mäechler, M., Magnusson, A., Möller, S., et al. (2005). Gplots: Various R Programming Tools for Plotting Data – ScienceOpen. https://www.scienceopen.com/document?vid=0e5d8e31-1fe4-492f-a3d8-8cd71b2b8ad9.

OMTON, Volume *32*

Supplemental information

Gut microbiota composition is associated with the efficacy of Delta-24-RGDOX in malignant gliomas

Natalie M. Meléndez-Vázquez, Teresa T. Nguyen, Xuejun Fan, Andrés R. López-Rivas, Juan Fueyo, Candelaria Gomez-Manzano, and Filipa Godoy-Vitorino

Supplemental Information

Table S1: Experimental groups, number of animals and summary of the number of sequences (reads) and ASVs across the GSC-005 glioblastoma cohort dataset.

	Experimental Group	Samples	Mice Survival Period	Mean Survival (days) ± Stdev*	Average Number of Reads ± Stdev*	Average Number of ASVs ± Stdev*
**GSC-005 Glioblastoma Cohort**	Naive	5	NA	NA	19432.00 ± 11259.68	209.00 ± 34.31
	PBS	3	50 - 56 days	51.33 ± 1.53	19725.00 ± 10266.22	290.67 ± 93.07
	Indoximod	5	50 - 56 days	53.40 ± 2.07	10415.20 ± 2436.14	203.60 ± 15.47
	Delta-24-RGDOX	4	More than 100 days	168.00 ± 39.23	27508.50 ± 14201.95	219.75 ± 68.65
	**TOTAL**	17				

*Stdev: standard deviation

Table S2: Experimental groups, number of animals and summary of the number of sequences (reads) and ASVs across the GL261-5 glioblastoma cohort dataset.

	Experimental Group	Samples	Mean Survival (days) ± Stdev*	Average Number of Reads ± Stdev*	Average Number of ASVs ± Stdev*
**GL261-5 Glioblastoma Cohort**	PBS	5	43.00 ± 6.00	15,536.80 ± 1417.51	176.80 ± 26.25
	Delta-24-RGDOX + Indoximod + αCD4	5	53.0 ± 18.60	14,807.20 ± 2098.82	180.60 ± 30.32
	Delta-24-RGDOX + Indoximod + IgG	5	100.0 ± 0.00	13,857.40 ± 1426.24	184.00 ± 18.68
	**TOTAL**	15			

*Stdev: standard deviation

Table S3: Statistical values of Tukey's Multiple Comparisons Test on the median survival of both GSC-005 and GL261-5 GBM cohorts.

	Experimental Group 1	Experimental Group 2	PERMANOVA p-value
**GSC-005 GBM Cohort**	PBS (51.33 days ± 1.53) (n=3)	Indoximod (53.40 days ± 2.07) (n=5)	0.9915
	PBS (51.33 days ± 1.53) (n=3)	Delta-24-RGDOX (168.00 days ± 39.23) (n=4)	**0.0002**
	Indoximod (53.40 days ± 2.07) (n=5)	Delta-24-RGDOX (168.00 days ± 39.23) (n=4)	**<0.0001**
**GL261-5 GBM Cohort**	PBS (43.00 days ± 6.00) (n=5)	Delta-24-RGDOX + Indoximod + αCD4 (53.0 days ± 18.60) (n=5)	0.371
	PBS (43.00 days ± 6.00) (n=5)	Delta-24-RGDOX + Indoximod + IgG (100.0 days ± 0.00) (n=5)	**<0.0001**
	Delta-24-RGDOX + Indoximod + αCD4 (53.0 days ± 18.60) (n=5)	Delta-24-RGDOX + Indoximod + IgG (100.0 days ± 0.00) (n=5)	**<0.0001**

Significant p-values in bold.

Table S4: Statistical values of PERMANOVA and Kruskal-Wallis (KW) pairwise tests on the compositional biplots and alpha diversity metrics for the main text Figure 2.

			Figure 2A	Figure 2B	Figure 2C	Figure 2D
	**Experimental Group 1**	**Experimental Group 2**	**PERMANOVA p-value**	**PERMANOVA p-value**	**Chao1 KW p-value**	**Shannon KW p-value**
**GSC-005 GBM Cohort**	Naive (n=5)	PBS 50-56 days (n=3)	**0.022**	-	0.051	**0.025**
	Naive (n=5)	Indoximod 50-56 days (n=5)	**0.009**	-	0.753	0.076
	Naive (n=5)	Delta-24-RGDOX more than 100 days (n=4)	**0.007**	-	0.806	0.806
	PBS 50-56 days (n=3)	Indoximod 50-56 days (n=5)	**0.017**	**0.017**	0.101	**0.025**
	PBS 50-56 days (n=3)	Delta-24-RGDOX more than 100 days (n=4)	0.051	**0.032**	0.289	0.157
	Indoximod 50-56 days (n=5)	Delta-24-RGDOX more than 100 days (n=4)	0.139	0.059	1.000	0.462

Significant p-values in bold.

Table S5: Statistical values of the Wilcoxon Rank-Sum (WRST) pairwise test on the Firmicutes/Bacteroidetes ratio for the main text Figure 3.

	Experimental Group 1	Experimental Group 2	WRST p-value
**GSC-005 GBM Cohort**	Naive (n=5)	PBS 50-56 days (n=3)	0.21
	Naive (n=5)	Indoximod 50-56 days (n=5)	1.00
	Naive (n=5)	Delta-24-RGDOX more than 100 days (n=4)	1.00
	PBS 50-56 days (n=3)	Indoximod 50-56 days (n=5)	0.36
	PBS 50-56 days (n=3)	Delta-24-RGDOX more than 100 days (n=4)	0.91
	Indoximod 50-56 days (n=5)	Delta-24-RGDOX more than 100 days (n=4)	0.91

Significant p-values in bold.

Table S6: Statistical values of PERMANOVA, Kruskal-Wallis (KW) and Wilcoxon Rank-Sum (WRST) pairwise tests on the compositional biplots, alpha diversity metrics, and F/B ratios respectively for main text Figure 5.

			**Figure 5A**	**Figure 5B**		**Figure 5C**
	**Experimental Group 1**	**Experimental Group 2**	**PERMANOVA p-value**	**Chao1 KW p-value**	**Shannon KW p-value**	**WRST p-value**
**GL261-5 GBM Cohort**	PBS (n=5)	Delta-24-RGDOX + Indoximod + αCD4 (n=5)	0.055	0.754	0.917	0.67
	PBS (n=5)	Delta-24-RGDOX + Indoximod + IgG (n=5)	**0.015**	0.465	0.754	0.67
	Delta-24-RGDOX + Indoximod + αCD4 (n=5)	Delta-24-RGDOX + Indoximod + IgG (n=5)	0.265	0.917	0.754	0.67

Significant p-values in bold.

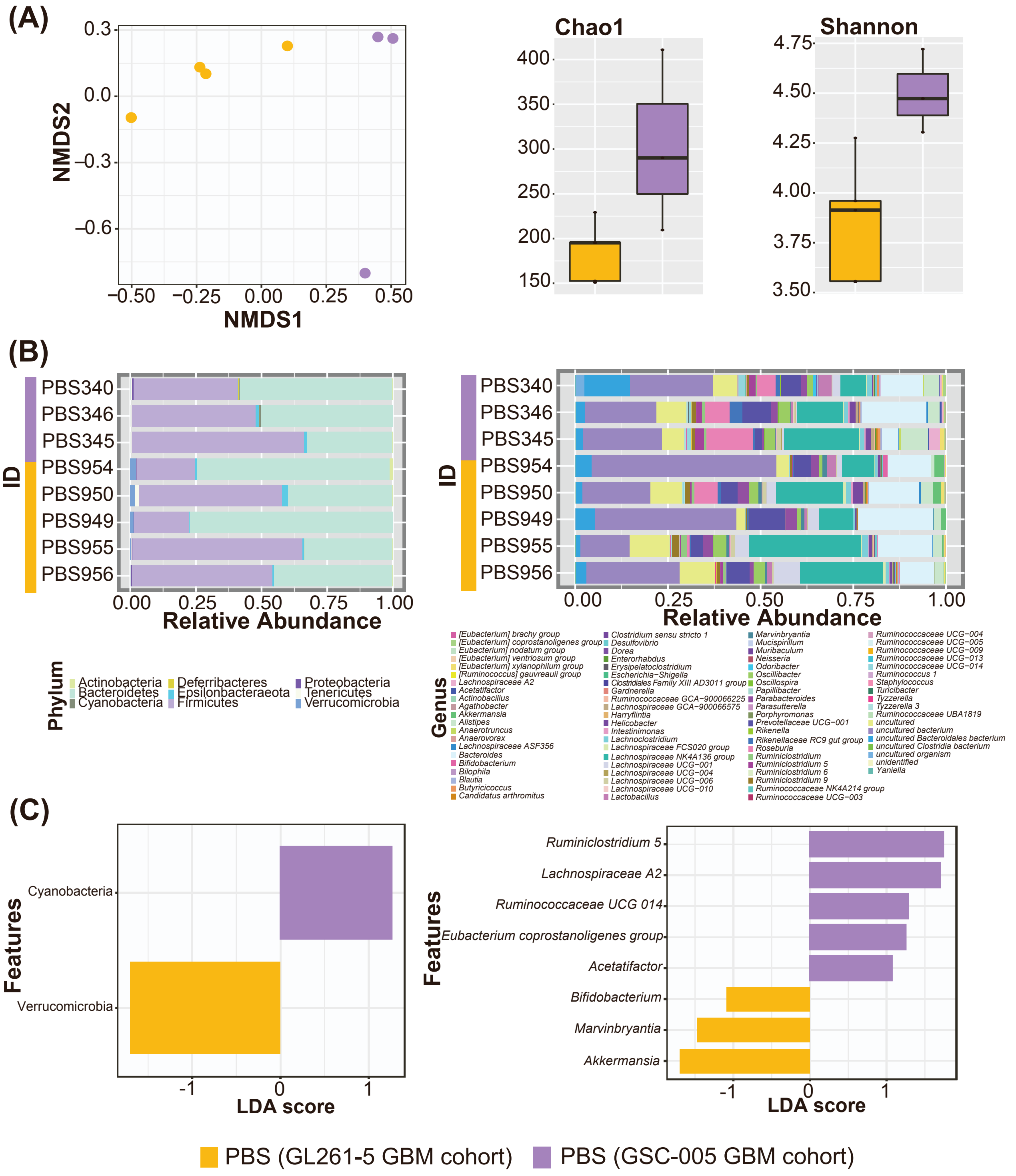

Figure S1: **(A)** Changes in bacterial composition and structure between the control groups (PBS administered mice) of the GL261-5 and GSC-005 glioma-bearing mice (ANOSIM p-value=0.016; stress value=9.572417e-05). Even though no differences in richness (Chao1) were observed (KW p-value=0.05), there is a tendency of a higher number of species in the PBS GSC-005 glioma-bearing mice. When evaluating the Shannon Index, we observed significant differences in the bacterial community diversity between both groups (KW p-value=0.03), observing a higher diversity in the PBS GSC-005 glioma-bearing mice. **(B)** Demonstrates the taxonomic changes at phylum and genus level between both groups. We observe a higher abundance of Verrucomicrobia in the GL261-5 glioma-bearing mice, while Cyanobacteria and Tenericutes is higher in GSC-005 glioma-bearing mice. **(C)** Highlights the biomarkers of each PBS group with a Linear Discriminant Analysis Effect Size (LeFSe) (Log LDA Score: 1.0; p-value cutoff: 0.05 Original). These differences highlight the importance of having separate microbiome analysis for both glioblastoma cohorts.

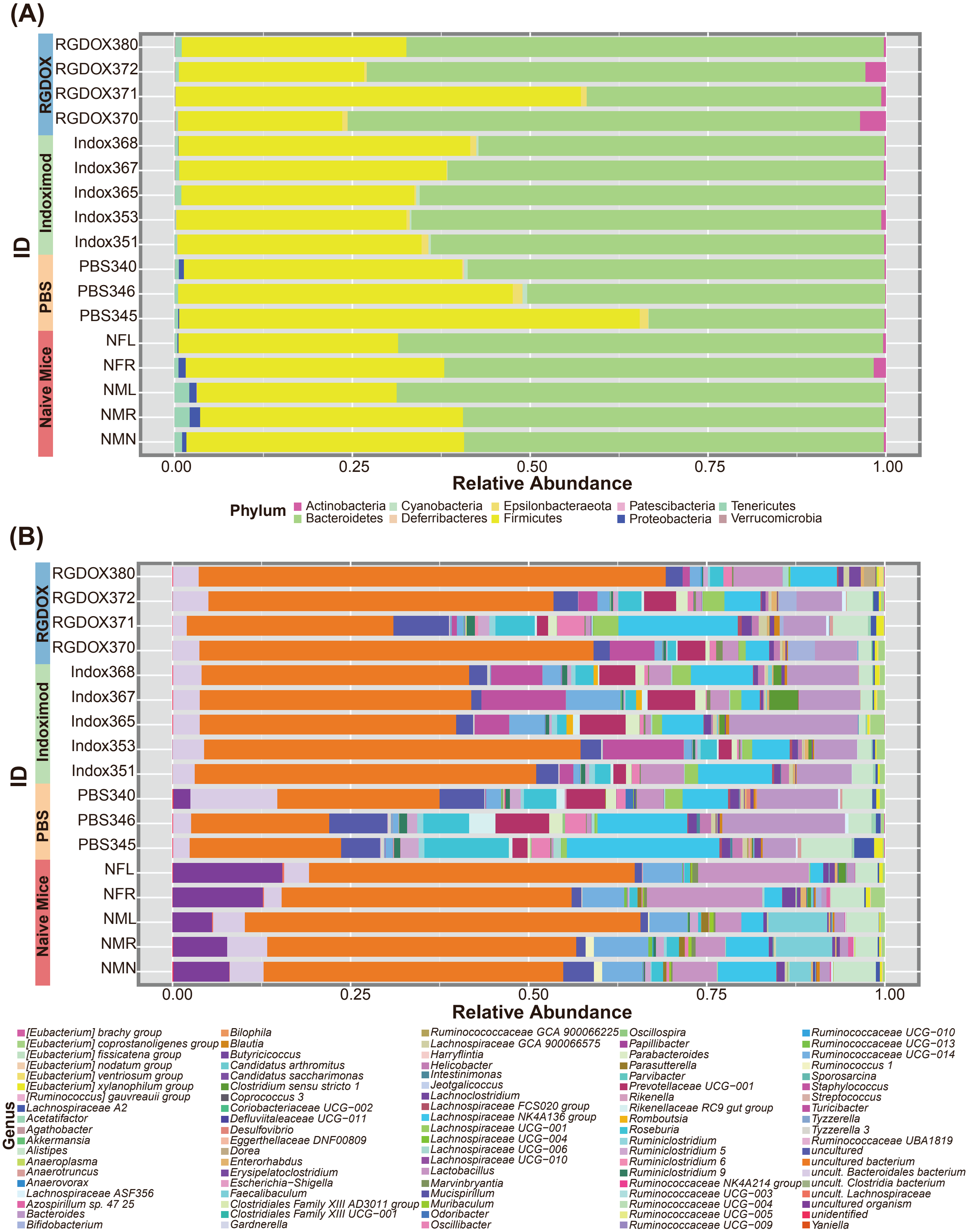

Figure S2: Taxonomic composition of gut bacteria between groups showing that responders to oncolytic viral therapy that had longer survival periods have a gut microbiome profile similar to that of Naive mice. Taxonomic changes are observed at phylum (A) and genus (B) level between groups. We observe a higher abundance of Actinobacteria in the oncolytic viral therapy mice responders, highlighted by an abundance of *Bifidobacterium*.

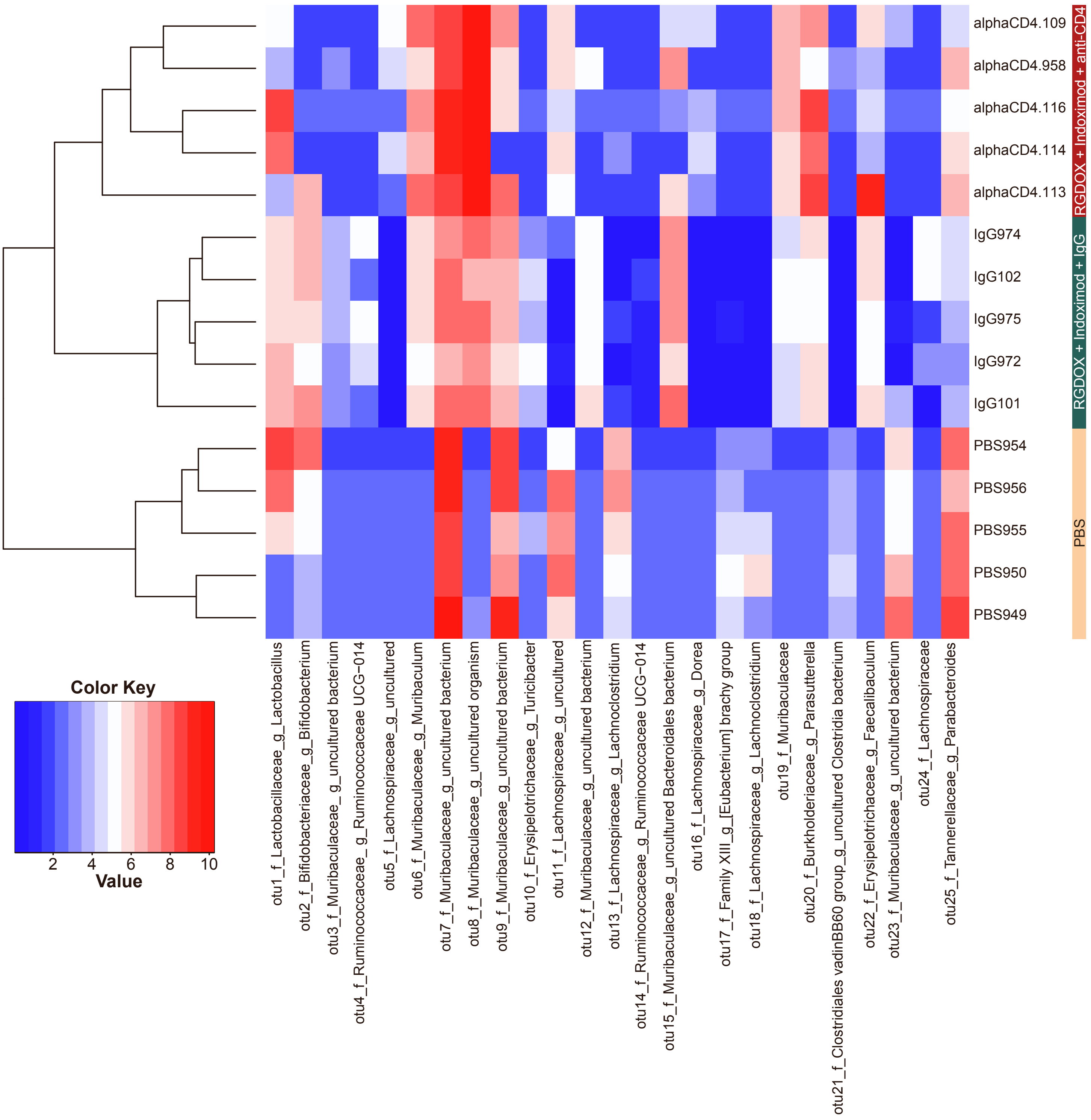

Figure S3: Heatmap of significant ASVs (raw p-value<0.01) from the GL261-5 cohort highlights the significant reduction of *Bifidobacterium* in the viroimmunotherapy and Indoximod-treated mice that had depleted $CD4^{+}$ T cells.
